# NLRP12 in cancer: a context-dependent regulator of tumor progression, immunity, and metabolism

**DOI:** 10.3389/fonc.2026.1862634

**Published:** 2026-07-10

**Authors:** Yuting Cen, Li Li, Xinyu Ding, Chenyang Wang, Hua Qiu

**Affiliations:** 1 Department of Traditional Chinese Medicine, Guangxi Medical University Cancer Hospital, Nannning, Guangxi, China; 2 Department of Traditional Chinese Medicine, The First Affiliated Hospital of Guangxi Medical University, Nannning, Guangxi, China

**Keywords:** cancer progression, context dependency, metabolic reprogramming, NLRP12, tumor immunity

## Abstract

NLRP12, a member of the NOD-like receptor family, has traditionally been regarded as an inflammasome-associated regulator of inflammatory signaling. However, accumulating evidence indicates that its role in cancer extends far beyond classical inflammasome biology. Recent studies show that NLRP12 exerts highly context-dependent functions across malignancies, acting as either a tumor suppressor or a tumor promoter depending on tumor type, cellular source, and dominant signaling environment. In inflammation-associated and epithelial malignancies such as colorectal cancer, hepatocellular carcinoma, and triple-negative breast cancer, NLRP12 suppresses tumor progression by restraining noncanonical NF-κB, Wnt/β-catenin, JNK, or canonical NF-κB signaling. In contrast, in gastric cancer, ovarian cancer, glioma, and macrophage-rich tumor ecosystems, NLRP12 has been linked to glycolytic remodeling, lactate-associated epigenetic adaptation, aggressive clinicopathological features, and immune suppression. Mechanistically, NLRP12 has emerged as a multifunctional signaling regulator that connects inflammatory control with oncogenic pathway modulation, metabolic rewiring, tumor-associated macrophage polarization, and PANoptosis-related stress responses. These findings position NLRP12 at the crossroads of tumor progression, immunity, metabolism, and inflammatory cell death. In this review, we summarize the molecular and functional landscape of NLRP12 in cancer, with emphasis on its dual roles in tumor biology, its context-specific mechanisms, and its potential clinical relevance as a biomarker and therapeutic reference point. A deeper cell-resolved and mechanism-oriented understanding of NLRP12 may help redefine this molecule from a conventional innate immune regulator to a context-dependent organizer of tumor ecosystems.

## Highlights

- NLRP12 exerts context-dependent tumor-suppressive or tumor-promoting roles in cancer.
- NLRP12 links inflammatory signaling with Wnt/JNK/NF-κB, metabolism, and immunity.
- NLRP12 is an emerging biomarker and therapeutic reference point in tumor ecosystems.

## 1 Introduction

Cancer development is no longer viewed as a process driven solely by genetic mutations within malignant cells (1–3). Instead, tumor initiation and progression are increasingly understood as dynamic outcomes of reciprocal interactions among oncogenic signaling, chronic inflammation, metabolic adaptation, and immune remodeling. These processes do not operate independently; rather, they converge to shape tumor cell fitness, stromal reprogramming, and therapeutic responsiveness (4, 5). In this context, molecules that couple inflammatory signaling to metabolic and immune states have attracted growing attention, because they may explain why tumors with distinct tissue origins or microenvironmental compositions exhibit markedly different biological behaviors.

Members of the nucleotide-binding oligomerization domain-like receptor (NLR) family are classically recognized as innate immune sensors that detect danger-associated signals and regulate inflammatory responses (6). Beyond their established roles in host defense and inflammasome biology, NLR proteins are now increasingly implicated in cancer-related processes, including epithelial transformation, tumor-promoting inflammation, immune escape, and therapy resistance (7). Depending on cellular context, NLR family members may function either as barriers against chronic inflammatory tumorigenesis or as facilitators of tumor-supportive microenvironmental adaptation (8). This conceptual expansion has made it necessary to reassess individual NLR molecules not only as immune sentinels, but also as broader regulators of tumor biology.

Among these molecules, NLRP12 has historically been characterized as a negative regulator of inflammatory signaling, particularly through suppression of NF-κB-associated pathways (9). Early evidence established NLRP12 as an important brake on intestinal inflammation and inflammation-associated tumorigenesis, especially through the negative regulation of noncanonical NF-κB signaling (10). This anti-inflammatory role initially supported the view that NLRP12 acts predominantly as a tumor suppressor in inflammation-driven malignancies. Subsequent work strengthened this interpretation by showing that NLRP12 can restrain hepatocarcinogenesis through suppression of c-Jun N-terminal kinase (JNK) activation and inhibit malignant progression in triple-negative breast cancer through repression of NF-κB signaling. In colorectal cancer, more recent mechanistic evidence has further extended the functional spectrum of NLRP12 by demonstrating that it can downregulate Wnt/β-catenin signaling through interaction with STK38, thereby suppressing tumor growth, epithelial-mesenchymal transition, and invasive progression. However, this tumor-suppressive framework no longer captures the full biological complexity of NLRP12 in cancer. Emerging studies indicate that NLRP12 may also exert tumor-promoting functions in specific malignancies or cellular compartments. In gastric cancer, NLRP12 has been reported to stabilize hexokinase 2 (HK2) by reducing TRIM25-mediated degradation, thereby enhancing glycolysis, lactate production, histone H3K18 lactylation, and MYC-driven transcriptional output (11). In ovarian cancer and glioma, elevated NLRP12 expression has been associated with aggressive clinicopathological features and poor prognosis (12). Moreover, in lung adenocarcinoma, NLRP12 expression in tumor-associated macrophages has been linked to immunosuppressive polarization through a C1qA/LILRB4/NF-κB-dependent circuit, suggesting that the biological role of NLRP12 cannot be interpreted solely from tumor cell-intrinsic studies. These observations collectively indicate that NLRP12 may function as either a tumor suppressor or a tumor promoter depending on tumor type, cellular source, dominant signaling environment, and microenvironmental context.

This context dependency is particularly important because it places NLRP12 at the intersection of several major hallmarks of cancer. First, NLRP12 participates in inflammatory signal regulation, including canonical and noncanonical NF-κB signaling as well as JNK-associated stress pathways. Second, it influences oncogenic signaling networks that directly control proliferation, differentiation, and invasion, exemplified by its regulation of the STK38/GSK3β/β-catenin axis in colorectal cancer. Third, NLRP12 appears capable of shaping tumor metabolism and epigenetic adaptation, as suggested by its role in glycolytic reprogramming and lactate-associated chromatin modification in gastric cancer. Fourth, NLRP12 contributes to tumor immune ecology by modulating macrophage behavior, immune infiltration patterns, and possibly broader immunosuppressive programs. Finally, recent work linking HCK to NLRP12-mediated PANoptosis suggests that NLRP12 may also influence inflammatory cell death programs with potential implications for tumor immunogenicity and treatment response (13). Taken together, these findings support a revised view of NLRP12 as a multifunctional and context-sensitive regulator rather than a unidirectional anti-inflammatory factor.

Despite this growing body of evidence, the cancer biology of NLRP12 remains insufficiently synthesized. Current studies are fragmented across tumor types and often emphasize either tumor-intrinsic signaling or immune-related functions without integrating both dimensions into a unified framework. In addition, major questions remain unresolved: why does NLRP12 restrain tumorigenesis in some settings but promote tumor progression in others? To what extent are its functions determined by malignant cells versus stromal or immune compartments? Is NLRP12 best interpreted as an inflammasome-related molecule, a signaling scaffold, a metabolic regulator, or a microenvironmental modulator? Addressing these questions is essential not only for mechanistic understanding but also for evaluating whether NLRP12 has realistic potential as a biomarker or therapeutic target.

In this review, we summarize current evidence on the role of NLRP12 in cancer, with emphasis on why its functions differ across tumor types and cellular compartments. We first outline the molecular features of NLRP12, then discuss its tumor-suppressive roles in colorectal cancer, hepatocellular carcinoma, and triple-negative breast cancer, as well as its tumor-promoting associations in gastric cancer, ovarian cancer, glioma, and macrophage-rich tumor microenvironments. We further examine how NLRP12 intersects with immune remodeling, metabolic adaptation, and PANoptosis-related signaling, before considering its clinical and therapeutic implications. By integrating these findings, this review aims to provide a concise framework for interpreting NLRP12 as a tumor- and cell-context-dependent factor in cancer biology.

## 2 Molecular and biological features of NLRP12

### 2.1 Structure and canonical functions of NLRP12

NLRP12 belongs to the NOD-like receptor (NLR) family and contains the canonical tripartite domain architecture shared by multiple NLRP members, including an N-terminal pyrin domain (PYD), a central nucleotide-binding and oligomerization NACHT domain, and C-terminal leucine-rich repeats (LRRs) (14). The PYD is generally involved in homotypic protein–protein interactions, particularly with adaptor proteins carrying a compatible death-fold domain, whereas the NACHT domain supports ATP-dependent conformational change and oligomerization, and the LRR region is thought to contribute to autoregulation, ligand responsiveness, and intramolecular restraint. Structural work on the NLRP12 PYD has shown that this domain adopts the conserved death-domain fold typical of inflammasome-related proteins, supporting its potential role in higher-order signaling assemblies (15).

NLRP12 was initially studied in the context of innate immunity and autoinflammatory disease, where it was largely regarded as a regulator of inflammatory signal transduction rather than a conventional oncogenic or tumor-suppressive molecule. Early work established that NLRP12 can function as a negative regulator of inflammatory pathways, particularly NF-κB-associated signaling (16, 17). In models of colitis and colitis-associated tumorigenesis, NLRP12 restrains noncanonical NF-κB activation by interacting with NF-κB-inducing kinase (NIK) and TRAF3, thereby limiting NIK accumulation, reducing p100 processing to p52, and suppressing transcription of downstream inflammatory and tumor-promoting mediators. In addition to its effects on noncanonical NF-κB signaling, NLRP12 has also been linked to suppression of canonical inflammatory signaling in several experimental systems. Reviews synthesizing this literature note that NLRP12 can dampen excessive inflammatory responses upstream of NF-κB activation and thereby act as a checkpoint against chronic tissue injury and inflammatory amplification. This anti-inflammatory role formed the conceptual basis for viewing NLRP12 as a barrier to inflammation-associated disease progression.

At the same time, NLRP12 cannot be reduced to a single inhibitory axis. Like several NLR proteins, it has also been discussed in the context of inflammasome-related biology, although its inflammasome-forming potential appears more restricted, context-specific, and less canonical than that of NLRP3. Recent work has further complicated this picture by showing that NLRP12 can interact with other inflammasome-related proteins and participate in broader inflammatory signaling networks rather than acting solely as a standalone inflammasome sensor. These observations suggest that the biological identity of NLRP12 is inherently multifunctional and should not be interpreted exclusively through the classical inflammasome paradigm.

Compared with other NLR family members, NLRP12 occupies a relatively distinctive position in cancer biology (6–8). NLRP3 is best known as a canonical inflammasome sensor that promotes caspase-1 activation and IL-1β/IL-18 maturation, and its role in cancer is often discussed in relation to inflammation-driven tumor progression, pyroptosis, and immune modulation (18, 19). NLRP1 and NLRC4 are also more closely associated with inflammasome assembly and inflammatory cell death, whereas NOD1 and NOD2 mainly function as intracellular pattern-recognition receptors that regulate microbial sensing and NF-κB/MAPK activation (20, 21). By contrast, NLRP12 appears to act less as a classical inflammasome-forming sensor and more as a context-dependent signaling regulator. Its cancer-related functions include suppression of noncanonical NF-κB signaling, regulation of Wnt/β-catenin and JNK pathways, modulation of metabolic enzyme stability, and reshaping of macrophage-associated immune suppression (11, 13). This broader scaffold-like and pathway-selective behavior may explain why NLRP12 displays particularly divergent tumor-suppressive or oncogenic roles across malignancies.

### 2.2 NLRP12 beyond inflammasome biology

This distinction from classical inflammasome-centered NLRs is important for understanding why NLRP12 should not be interpreted solely through the lens of inflammasome activation. Accumulating evidence now indicates that the functional repertoire of NLRP12 extends well beyond inflammasome-associated inflammatory control. In cancer and other disease settings, NLRP12 behaves increasingly like a context-sensitive signaling regulator capable of influencing transcriptional programs, kinase cascades, metabolic pathways, and cell death responses (22, 23). This broader view is essential for understanding why NLRP12 can exhibit divergent roles across different tumor types. A major conceptual advance came from studies showing that NLRP12 directly regulates oncogenic signaling circuits not classically associated with inflammasomes. In colorectal cancer, NLRP12 suppresses tumor progression by interacting with STK38 and reducing GSK3β phosphorylation, thereby promoting β-catenin degradation and attenuating Wnt/β-catenin pathway activation. This finding is particularly important because it places NLRP12 upstream of one of the most central signaling axes in epithelial tumorigenesis and indicates that NLRP12 can directly modulate tumor cell–intrinsic fate decisions, proliferation programs, and invasive behavior.

NLRP12 also regulates stress-associated kinase signaling outside the traditional inflammasome framework. In hepatocellular carcinoma, NLRP12 suppresses tumor development by downregulating JNK activation in hepatocytes, which in turn limits cJun and cMyc induction while preserving tumor-restrictive signals such as p21 (24). Conceptually, this expands the functional map of NLRP12 from an innate immune modulator to a guardian against inflammatory-proliferative kinase signaling in parenchymal tumor cells (25). More recent work has further broadened the field by linking NLRP12 to metabolic regulation and epigenetic adaptation. In gastric cancer, NLRP12 stabilizes HK2 by interfering with TRIM25-mediated degradation, thereby enhancing glycolysis, lactate production, and histone H3K18 lactylation, with downstream reinforcement of MYC-dependent transcriptional programs. Although this mechanism was defined in one tumor type, it suggests that NLRP12 may influence cancer biology not only through inflammatory signaling, but also through metabolic enzyme homeostasis and metabolite-driven chromatin remodeling. This represents a major departure from the view of NLRP12 as merely a suppressor of NF-κB activity.

Another emerging dimension is the role of NLRP12 in inflammatory cell death control. Recent PANoptosis-related literature has placed NLRP12 within multiprotein signaling assemblies that integrate pyroptotic, apoptotic, and necroptotic components (26–29). The identification of HCK as a regulator of NLRP12-mediated PANoptosis further supports the notion that NLRP12 can function as part of a broader stress-response platform influencing cell death sensitivity and inflammatory output. In the cancer context, this may have important implications for treatment response, tumor immunogenicity, and microenvironmental remodeling, even though these questions remain insufficiently explored.

**Figure 1:**
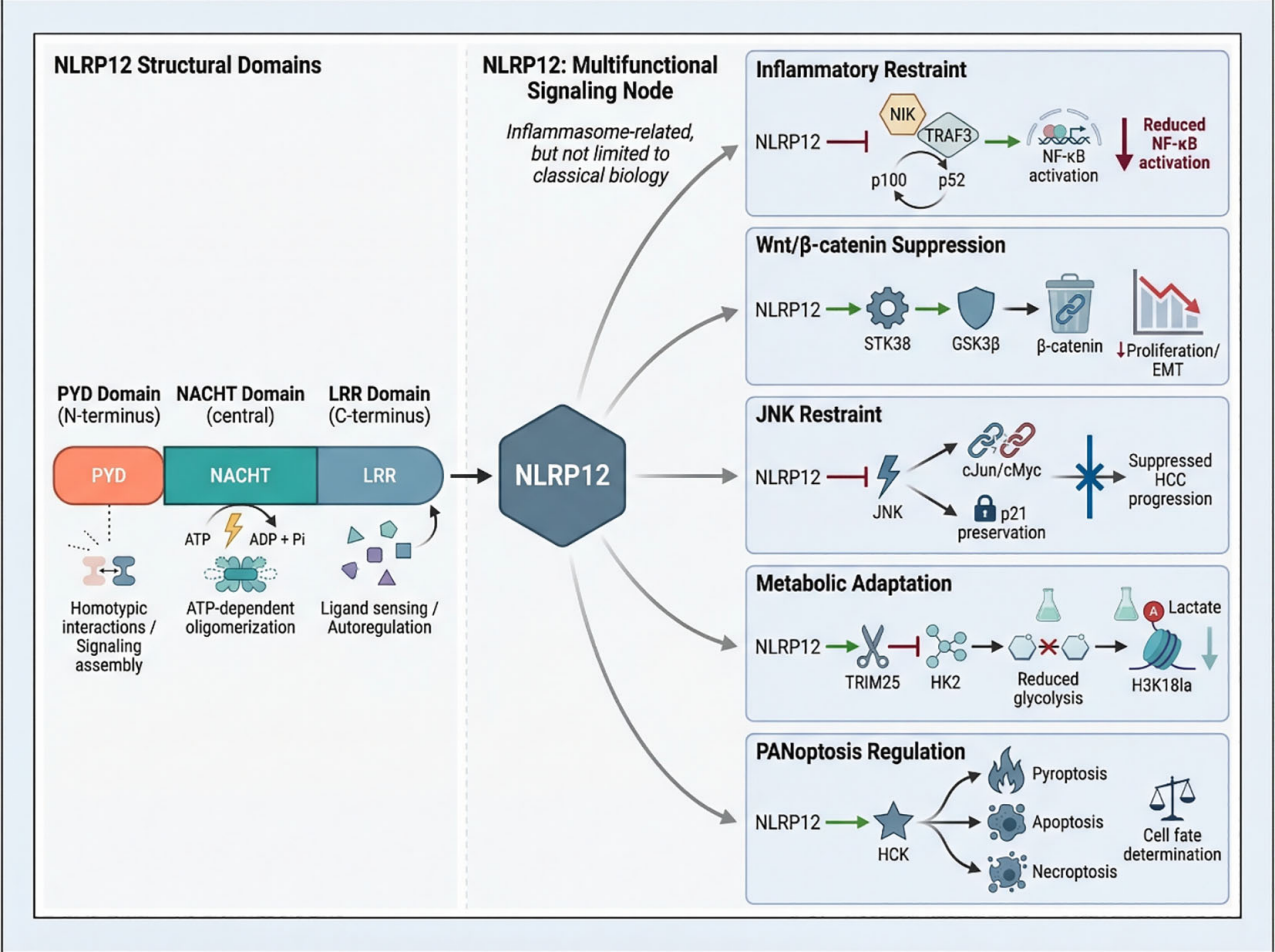
Structural domains and multifunctional signaling roles of NLRP12 in cancer. NLRP12 contains PYD, NACHT, and LRR domains and functions as a context-sensitive signaling node beyond classical inflammasome biology. It restrains inflammatory NF-κB signaling, suppresses Wnt/β-catenin and JNK pathways, regulates HK2-dependent glycolysis and H3K18 lactylation, and participates in HCK-associated PANoptosis-related signaling.

Taken together, these findings argue that NLRP12 should be viewed as a multifunctional signaling node rather than a single-purpose inflammasome component. Its activities span inflammatory restraint, oncogenic pathway modulation, metabolic rewiring, and cell death regulation, which collectively provide a mechanistic basis for its highly variable functions across cancers. **Figure 1** summarizes the structural organization of NLRP12 and highlights its multifunctional roles beyond classical inflammasome biology. As a context-sensitive signaling node, NLRP12 integrates inflammatory restraint, oncogenic pathway modulation, metabolic rewiring, and PANoptosis-related stress responses across cancer settings.

### 2.3 Determinants of context dependency

One of the most striking features of the current literature is that NLRP12 does not exert a uniform effect across malignancies. Instead, it acts as a tumor suppressor in some settings and a tumor promoter in others. This apparent contradiction is unlikely to be random; rather, it suggests that NLRP12 is governed by a set of context-defining determinants that shape its biological output. Specifically, the functional switch of NLRP12 appears to be determined by four major factors: tumor lineage-specific signaling dependency, cellular source of NLRP12 expression, local inflammatory–metabolic conditions, and the dominance of specific downstream pathways.

The first determinant is tumor lineage-specific signaling dependency. Different cancers rely on distinct dominant pathways for growth and survival, and NLRP12 may be functionally wired into these pathways in different ways. In colorectal cancer, where Wnt/β-catenin signaling is a core driver, NLRP12 constrains tumor progression by weakening β-catenin activation. In inflammation-associated colon tumorigenesis, its major role lies in suppressing noncanonical NF-κB signaling (30). In hepatocellular carcinoma, by contrast, its protective function appears to center on JNK restraint. These examples suggest that NLRP12 does not impose a universal program, but instead modifies the dominant vulnerability or dependency of the tissue-specific tumor context.

The second determinant is the cellular compartment in which NLRP12 is expressed. Tumor-cell-intrinsic NLRP12 and stromal/immune-cell-associated NLRP12 may have fundamentally different biological consequences. In malignant epithelial or parenchymal cells, NLRP12 more commonly functions as a signaling checkpoint that restrains oncogenic pathway activation and inflammatory amplification, as illustrated by its suppression of Wnt/β-catenin signaling in colorectal cancer, JNK signaling in hepatocellular carcinoma, and NF-κB signaling in triple-negative breast cancer. In contrast, when NLRP12 is expressed in stromal or immune compartments, particularly tumor-associated macrophages, it may support immunosuppressive polarization, weaken antitumor T-cell activity, and indirectly promote tumor progression (31, 32). Therefore, the net effect of NLRP12 in a given tumor is not determined by expression level alone, but by its dominant cellular source and the balance between tumor-cell-intrinsic and microenvironmental functions.

The third determinant is the microenvironmental inflammatory and metabolic state. Thus, an inflammation-dominant niche may favor the tumor-suppressive activity of NLRP12, whereas a lactate-rich, nutrient-stressed, or myeloid-enriched niche may shift NLRP12 toward tumor-promoting metabolic or immunosuppressive functions. NLRP12 is inherently positioned at the intersection of inflammatory signaling and tissue adaptation. In a tumor microenvironment characterized by chronic cytokine exposure, altered microbiota-derived signals, nutrient stress, lactate accumulation, or persistent myeloid activation, NLRP12 may be recruited into qualitatively different regulatory circuits. This may help explain why NLRP12 suppresses inflammation-driven tumorigenesis in some tissues yet contributes to glycolytic adaptation or immune suppression in others. Rather than acting in isolation, NLRP12 appears to respond to and reshape the local signaling ecology in which it is embedded. The fourth determinant is pathway dominance and signaling crosstalk. NLRP12 can influence NF-κB, Wnt/β-catenin, JNK, metabolic enzyme stability, and PANoptosis-related networks, but the relative importance of each axis is unlikely to be constant. In one setting, its most consequential action may be suppression of inflammatory transcription; in another, regulation of β-catenin turnover or glycolytic flux may dominate. This flexibility may reflect scaffold-like properties, differential binding partners, or post-translational regulation that shifts NLRP12 toward distinct signaling outputs.

Collectively, these determinants provide a conceptual framework for interpreting NLRP12 as a context-dependent regulator rather than a molecule with a fixed biological identity. In this framework, NLRP12 is more likely to act as a tumor suppressor when it restrains the dominant oncogenic inflammatory pathway, but may become oncogenic when it is recruited into metabolic adaptation, myeloid immunosuppression, or tumor-supportive pathway crosstalk. This perspective is essential for cancer research because it prevents oversimplified classification of NLRP12 as either uniformly protective or uniformly harmful. It also has direct translational implications: any attempt to use NLRP12 as a biomarker or therapeutic target will likely require precise definition of tumor lineage, cellular source, and dominant signaling environment.

## 3 Tumor-suppressive roles of NLRP12 in cancer

Although recent literature has revealed marked heterogeneity in the biological functions of NLRP12 across malignancies, some of the strongest mechanistic evidence still supports a tumor-suppressive role in specific epithelial and inflammation-associated cancers. In these contexts, NLRP12 functions as a regulatory checkpoint that restrains inflammatory signaling, limits oncogenic pathway activation, and suppresses proliferative or invasive transformation (33). Importantly, this suppressive activity is not mediated through a single universal mechanism. Instead, NLRP12 appears to engage different downstream pathways depending on tissue context, including noncanonical NF-κB signaling in colitis-associated tumorigenesis, Wnt/β-catenin signaling in colorectal cancer, JNK signaling in hepatocellular carcinoma, and canonical NF-κB signaling in triple-negative breast cancer (34). Collectively, these studies establish NLRP12 as a bona fide tumor suppressor in several settings, while also illustrating the pathway-selective nature of its protective functions.

### 3.1 NLRP12 restrains inflammation-associated colorectal tumorigenesis through negative regulation of noncanonical NF-κB signaling

The foundational evidence for a tumor-suppressive role of NLRP12 in cancer came from studies of intestinal inflammation and colitis-associated colorectal tumorigenesis. In the landmark Immunity study, Nlrp12-deficient mice were markedly more susceptible to colitis and colitis-associated colon cancer, indicating that NLRP12 acts as a critical checkpoint during chronic intestinal inflammation and tumor development. Mechanistically, this phenotype was linked to dysregulated noncanonical NF-κB signaling, with Nlrp12-deficient tumors showing increased NIK activity, enhanced p100 processing to p52, reduced TRAF3 stability, and upregulation of protumorigenic target genes such as Cxcl12 and Cxcl13. The study also found altered ERK and AKT activation in affected tissues, suggesting that loss of NLRP12 amplifies multiple cancer-associated signaling outputs downstream of inflammatory dysregulation (35–37).

**Figure 2:**
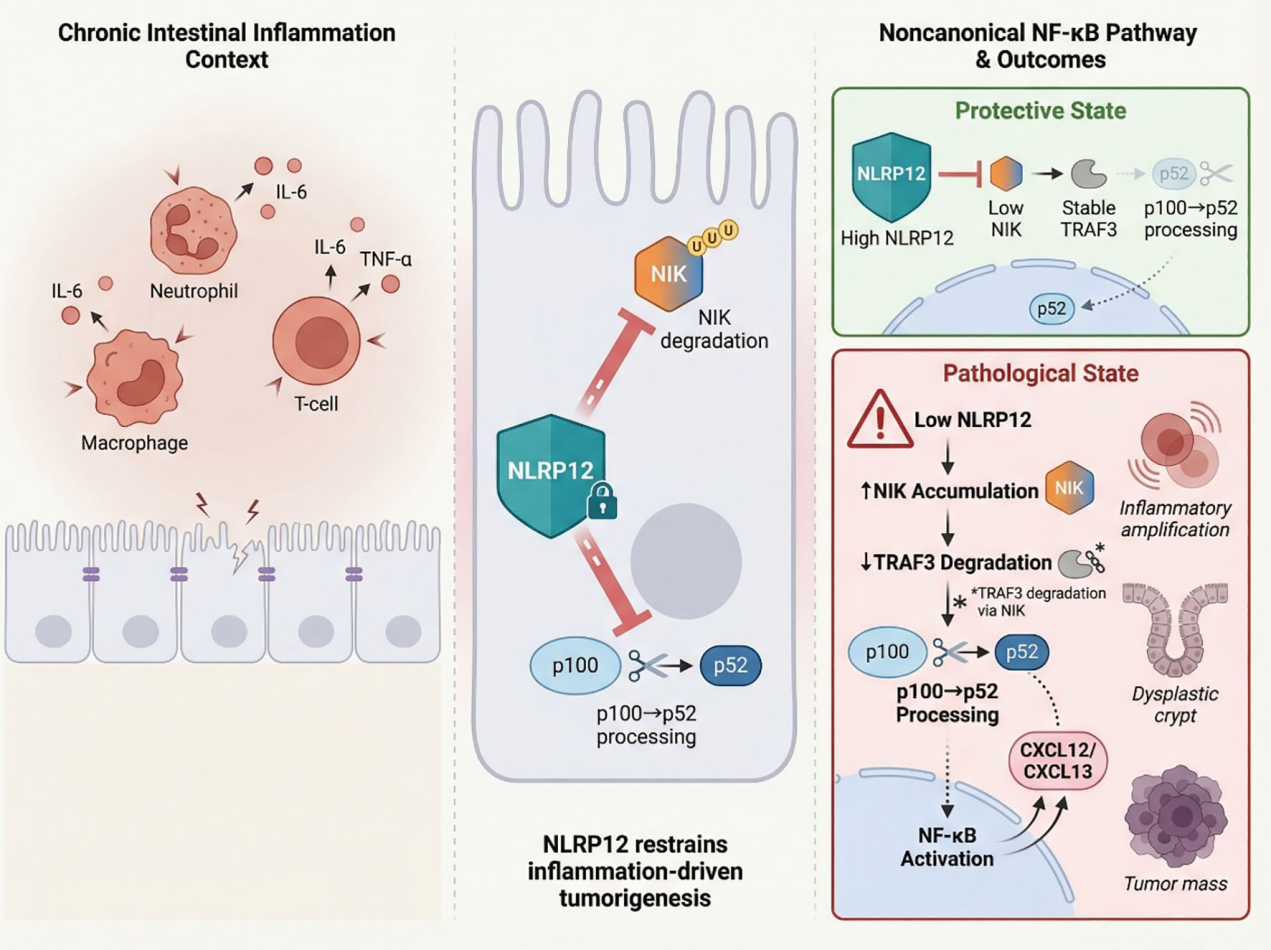
NLRP12 suppresses inflammation-associated colorectal tumorigenesis by negatively regulating noncanonical NF-κB signaling. NLRP12 restrains NIK accumulation, limits p100-to-p52 processing, and suppresses protumorigenic inflammatory mediators such as CXCL12 and CXCL13, thereby protecting the intestinal niche from chronic inflammatory amplification, epithelial transformation, and tumorigenesis.

An important conceptual contribution of this work is that it positioned NLRP12 not merely as a suppressor of acute inflammation, but as a molecular barrier against inflammation-driven tumorigenesis. The authors further showed that both hematopoietic and nonhematopoietic compartments contribute to disease control, although the nonhematopoietic compartment played a dominant role in tumorigenesis itself. This observation is especially relevant for later cancer studies because it implies that the antitumor effects of NLRP12 can arise from tissue-resident epithelial or stromal regulation rather than solely from immune-cell-intrinsic signaling. In this setting, NLRP12 appears to protect the intestinal niche by limiting the transition from chronic inflammatory injury to tumor-promoting signaling and epithelial transformation. **Figure 2** illustrates how NLRP12 functions as a protective checkpoint in the intestinal niche by restraining noncanonical NF-κB signaling and limiting the transition from chronic inflammation to colorectal tumorigenesis.

### 3.2 NLRP12 suppresses colorectal cancer progression via the STK38/GSK3β/β-catenin axis

More recent work has significantly expanded the tumor-suppressive framework of NLRP12 in colorectal cancer beyond inflammatory regulation alone. In a 2023 study published in the Journal of Clinical Investigation, NLRP12 was shown to suppress colorectal cancer by directly downregulating Wnt/β-catenin signaling through interaction with STK38 (38). Using conditional knockout mice, intestinal organoids, and colorectal cancer cells, the authors demonstrated that loss of NLRP12 enhanced β-catenin activation and cell proliferation, whereas intact NLRP12 constrained this oncogenic pathway in intestinal epithelial cells. Signaling pathway analysis revealed that Wnt/β-catenin activation, rather than NF-κB or MAPK signaling, was preferentially elevated in Nlrp12-deficient tumors, highlighting a distinct mechanism from earlier inflammation-centered studies (39–43).

**Figure 3:**
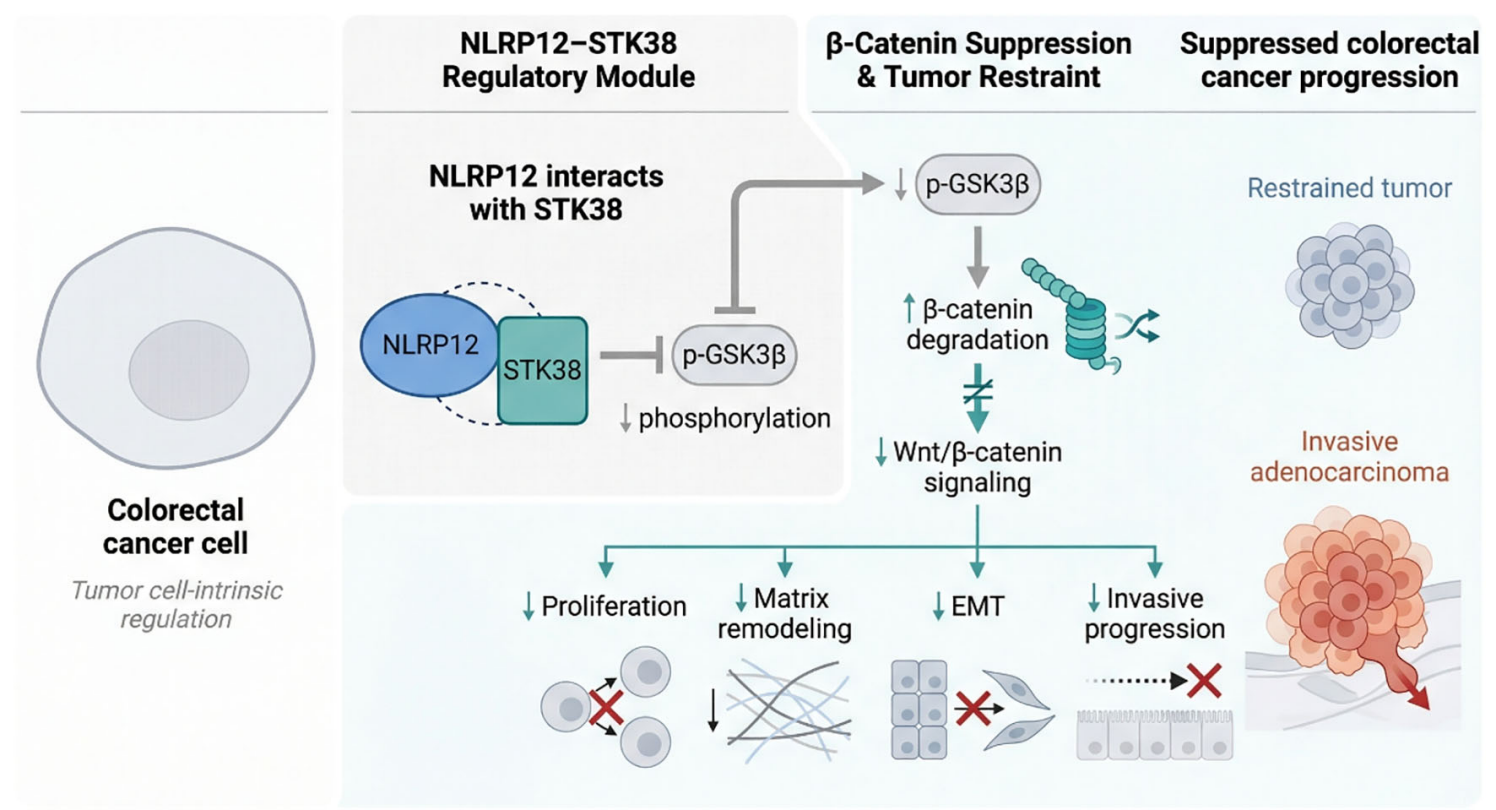
NLRP12 suppresses colorectal cancer progression through the STK38/GSK3β/β-catenin axis. By interacting with STK38, NLRP12 reduces GSK3β phosphorylation, promotes β-catenin degradation, and attenuates Wnt/β-catenin signaling, thereby limiting proliferation, matrix remodeling, epithelial–mesenchymal transition, and invasive progression in colorectal cancer.

Mechanistically, NLRP12 interacted with STK38 and reduced phosphorylation of GSK3β, thereby favoring β-catenin degradation and suppressing transcriptional programs associated with proliferation, matrix remodeling, and epithelial–mesenchymal transition. This is a major advance in the field because it demonstrates that NLRP12 can function as a direct regulator of a canonical epithelial oncogenic pathway rather than only as an upstream inflammatory brake. The consequences of NLRP12 loss were not limited to increased tumor burden; Nlrp12-deficient tumors also showed features of more invasive adenocarcinoma development, including elevated expression of genes linked to proliferation, matrix degradation, and EMT. Thus, in colorectal cancer, NLRP12 acts not only as a suppressor of tumor initiation, but also as a barrier to malignant progression and invasive transformation. Taken together, the 2012 Immunity study and the 2023 JCI study suggest that the protective role of NLRP12 in colorectal tumorigenesis is mechanistically layered. In inflammation-associated settings, NLRP12 restrains noncanonical NF-κB signaling and the inflammatory conditions that support cancer initiation. In tumor cell–intrinsic or epithelial contexts, it can also directly attenuate Wnt/β-catenin signaling through the STK38/GSK3β axis. These findings help explain why NLRP12 deficiency has such a strong impact on colorectal cancer phenotypes and underscore the versatility of its tumor-suppressive function in the intestinal epithelium. **Figure 3** illustrates the tumor cell-intrinsic mechanism by which NLRP12 restrains colorectal cancer progression through STK38-dependent suppression of β-catenin signaling.

### 3.3 NLRP12 suppresses hepatocellular carcinoma by restraining JNK-dependent hepatocyte proliferation and inflammation

**Figure 4:**
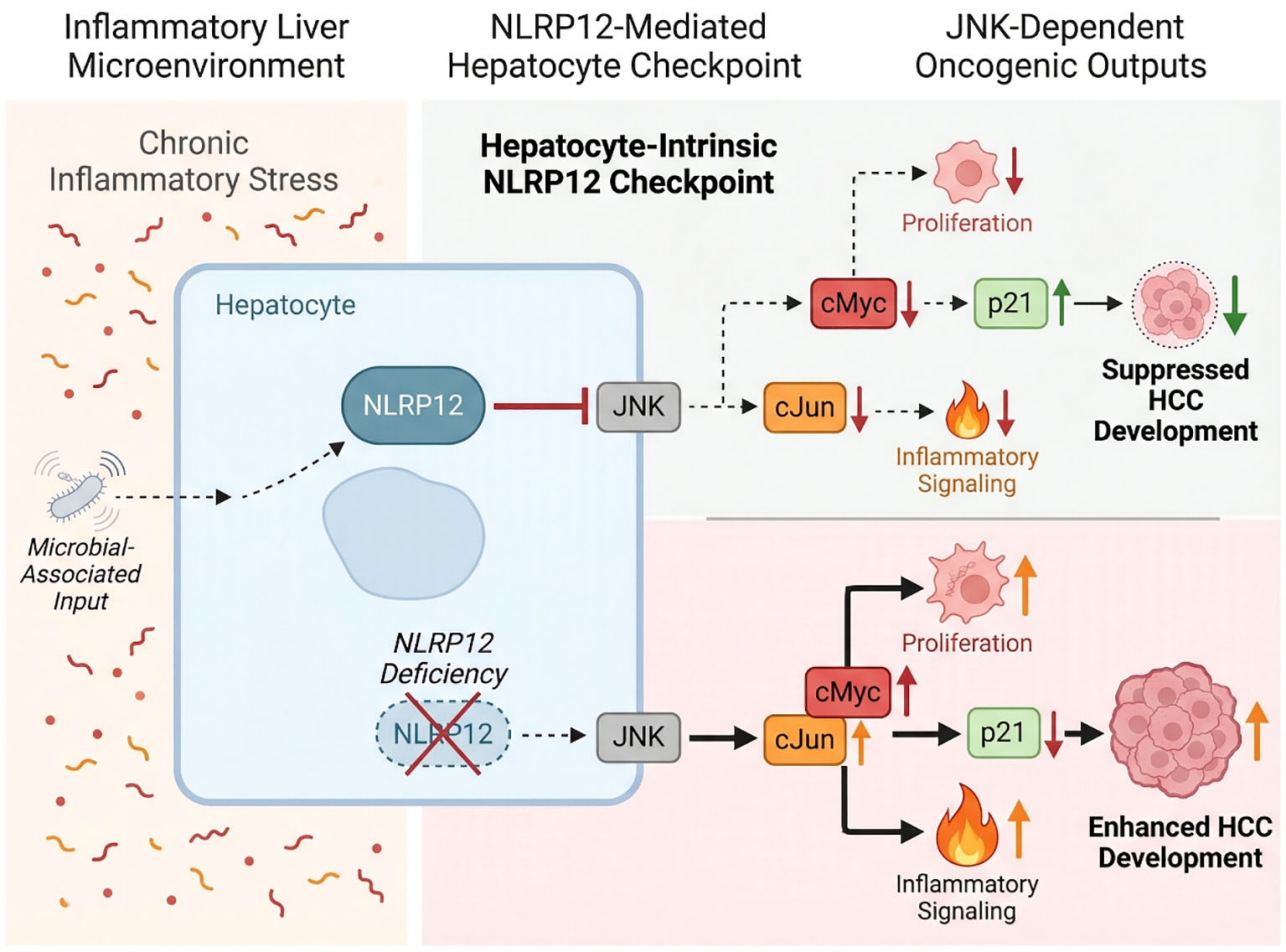
NLRP12 suppresses hepatocellular carcinoma by restraining JNK-dependent hepatocyte proliferation and inflammation. In hepatocytes, NLRP12 inhibits JNK activation, reduces cJun and cMyc expression, preserves p21, and limits inflammatory-proliferative signaling, thereby suppressing hepatocarcinogenesis. Microbial-associated inflammatory cues may further exacerbate tumor development when NLRP12 is lost.

A similarly compelling tumor-suppressive role for NLRP12 has been described in hepatocellular carcinoma (44). In the eLife study by Udden and colleagues, NLRP12 was identified as a negative regulator of HCC pathogenesis in hepatocytes. Using a diethylnitrosamine-driven liver tumor model, the authors showed that Nlrp12 deficiency promoted liver tumor development and was associated with enhanced inflammatory and proliferative signaling. Mechanistically, the dominant pathway altered in this setting was JNK, rather than NF-κB. Nlrp12-deficient tumors showed increased JNK activation along with higher expression of the proto-oncogenes cJun and cMyc and reduced expression of the tumor suppressor p21, supporting the conclusion that NLRP12 suppresses hepatocarcinogenesis through negative control of JNK-driven inflammation and proliferation. This study is particularly important because it shifts the focus of NLRP12 biology from mucosal inflammation to parenchymal tumor suppression. Rather than acting primarily through immune-cell-mediated inflammatory circuits, NLRP12 in hepatocytes appeared to directly constrain mitogenic and stress-associated signaling. The study also reported that antibiotic treatment attenuated the enhanced tumorigenesis seen in Nlrp12-deficient mice, suggesting that microbial or microbe-associated inflammatory cues contribute to the pathological consequences of NLRP12 loss. This finding reinforces a broader theme in NLRP12 biology: its tumor-suppressive effects often emerge at the interface of tissue-intrinsic signaling and inflammatory environmental input. In liver cancer, therefore, NLRP12 can be understood as a hepatocyte-intrinsic checkpoint that limits the conversion of chronic inflammatory stress into sustained oncogenic proliferation (**Figure 4**).

### 3.4 NLRP12 inhibits NF-κB-driven malignant behavior in triple-negative breast cancer

**Figure 5:**
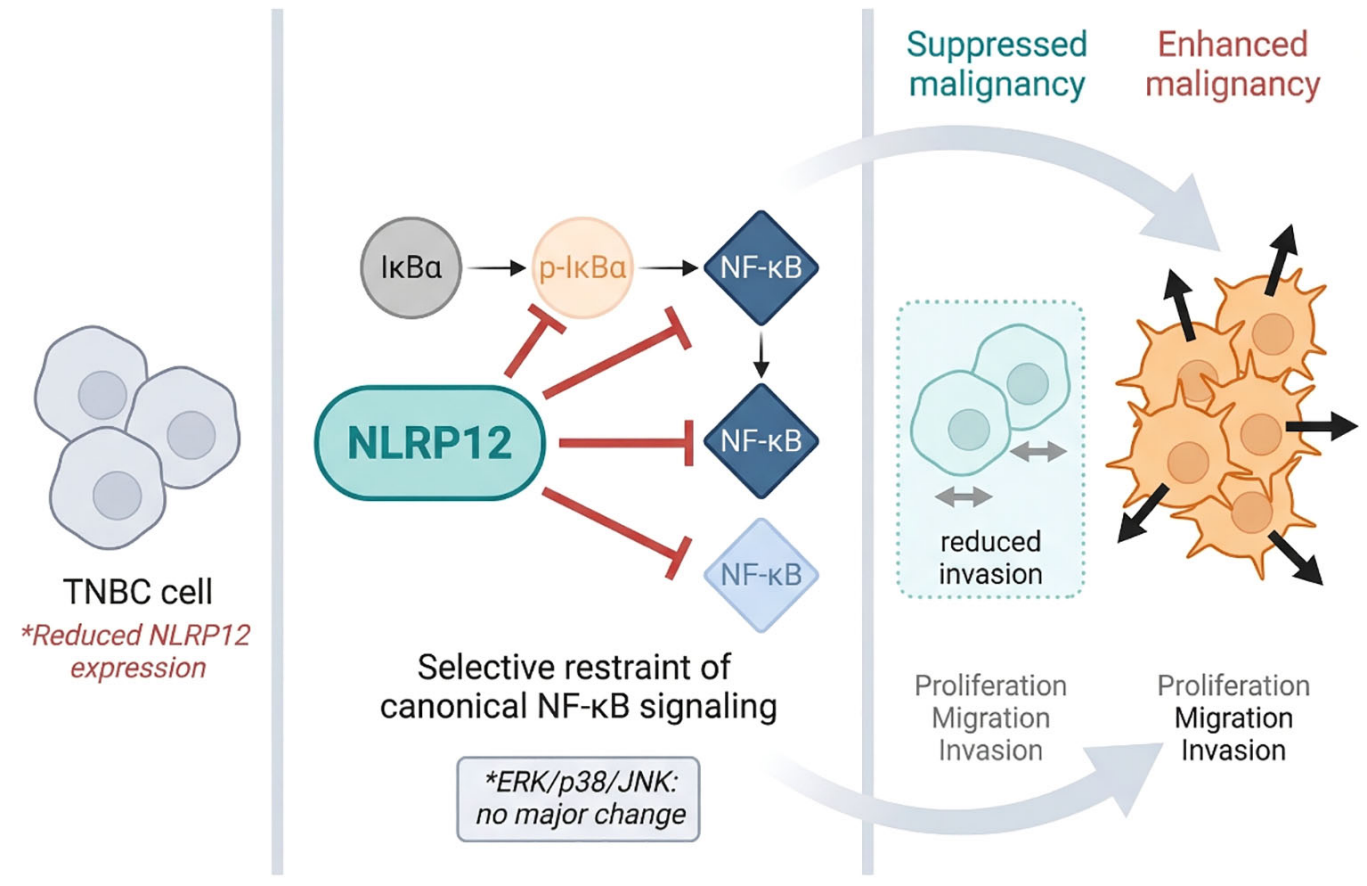
NLRP12 inhibits NF-κB-driven malignant behavior in triple-negative breast cancer. Reduced NLRP12 expression promotes IκBα phosphorylation and NF-κB activation, leading to enhanced proliferation, migration, and invasion, whereas intact NLRP12 restrains canonical NF-κB signaling and suppresses aggressive TNBC phenotypes.

Evidence supporting a tumor-suppressive role of NLRP12 has also emerged in triple-negative breast cancer, although the mechanistic depth is more limited than in colorectal cancer or hepatocellular carcinoma. In TNBC tissues, NLRP12 expression was reported to be reduced relative to nonmalignant controls, and experimental suppression of NLRP12 in MDA-MB-231 and MDA-MB-157 cells enhanced proliferation, migration, and invasion (45–47). The study further showed that NLRP12 silencing increased phosphorylated IκBα without significantly altering ERK, p38, or JNK signaling, indicating that NF-κB was the principal pathway activated by loss of NLRP12 in this model. Consistent with this interpretation, pharmacologic inhibition of NF-κB reversed the prometastatic effects induced by NLRP12 downregulation, and xenograft experiments supported the *in vitro* observations. Although this TNBC study is not as mechanistically comprehensive as the colorectal and liver cancer papers, it provides useful evidence that NLRP12 can suppress malignant behavior in breast cancer through selective restraint of canonical NF-κB signaling. It also reinforces a recurring pattern in the literature: when NLRP12 acts as a tumor suppressor, it often does so by buffering the signaling pathways that connect inflammatory activation to proliferation, survival, migration, and invasion (48). In this regard, the TNBC data fit well within the broader concept of NLRP12 as a context-dependent but frequently protective regulator in cancers where NF-κB-associated plasticity supports aggressive phenotypes (**Figure 5**).

### 3.5 A unifying view of the tumor-suppressive functions of NLRP12

Across colorectal cancer, colitis-associated tumorigenesis, hepatocellular carcinoma, and triple-negative breast cancer, several common themes emerge. First, NLRP12 consistently acts as a brake on signaling pathways that couple inflammatory stress to malignant progression. Second, its protective role is highly pathway-specific: noncanonical NF-κB predominates in colitis-associated cancer, Wnt/β-catenin in colorectal cancer, JNK in hepatocellular carcinoma, and canonical NF-κB in TNBC (49–52). Third, these studies collectively show that NLRP12 can function both in tissue-resident tumor cells and in nonhematopoietic compartments to suppress tumor-promoting reprogramming.

Overall, studies in colorectal cancer, hepatocellular carcinoma, and triple-negative breast cancer support a tumor-suppressive role for NLRP12. However, this protection is pathway-specific rather than universal: NLRP12 restrains noncanonical NF-κB signaling in colitis-associated tumorigenesis, Wnt/β-catenin signaling in colorectal cancer, JNK signaling in hepatocellular carcinoma, and canonical NF-κB signaling in TNBC. These examples show that NLRP12 suppresses tumor progression mainly by limiting the dominant tumor-promoting pathway in each tissue context.

## 4 Tumor-promoting roles of NLRP12 in specific cancer contexts

While a substantial body of work supports a tumor-suppressive role for NLRP12 in inflammation-associated colorectal tumorigenesis, hepatocellular carcinoma, and triple-negative breast cancer, more recent evidence has made it clear that this is not the whole story. In several tumor settings, NLRP12 expression correlates with aggressive clinicopathological features, poor prognosis, immunosuppressive microenvironmental remodeling, or enhanced metabolic adaptation. These studies challenge any attempt to classify NLRP12 as a uniformly protective molecule and instead support a more nuanced model in which its function is determined by tumor lineage, cellular source, and dominant signaling circuitry. Particularly notable are data from gastric cancer, ovarian cancer, and glioma, which together suggest that NLRP12 can be co-opted to support malignant progression under specific biological conditions.

### 4.1 NLRP12 promotes glycolytic remodeling and lactylation-driven transcriptional output in gastric cancer

Among the currently available tumor-promoting mechanisms, the evidence from gastric cancer is the most mechanistically developed. In a 2025 study, NLRP12 was shown to exert a significant cancer-promoting effect by stabilizing hexokinase 2 (HK2), a key enzyme in glycolytic flux (53). Mechanistically, NLRP12 competed with HK2 for binding to the E3 ligase TRIM25 and selectively reduced TRIM25-mediated K63-linked ubiquitination of HK2. As a result, HK2 degradation through the autophagosome–lysosome pathway was inhibited, leading to increased HK2 protein stability. This shift enhanced glycolysis and lactate production, placing NLRP12 upstream of a metabolic program that actively supports tumor progression.

**Figure 6:**
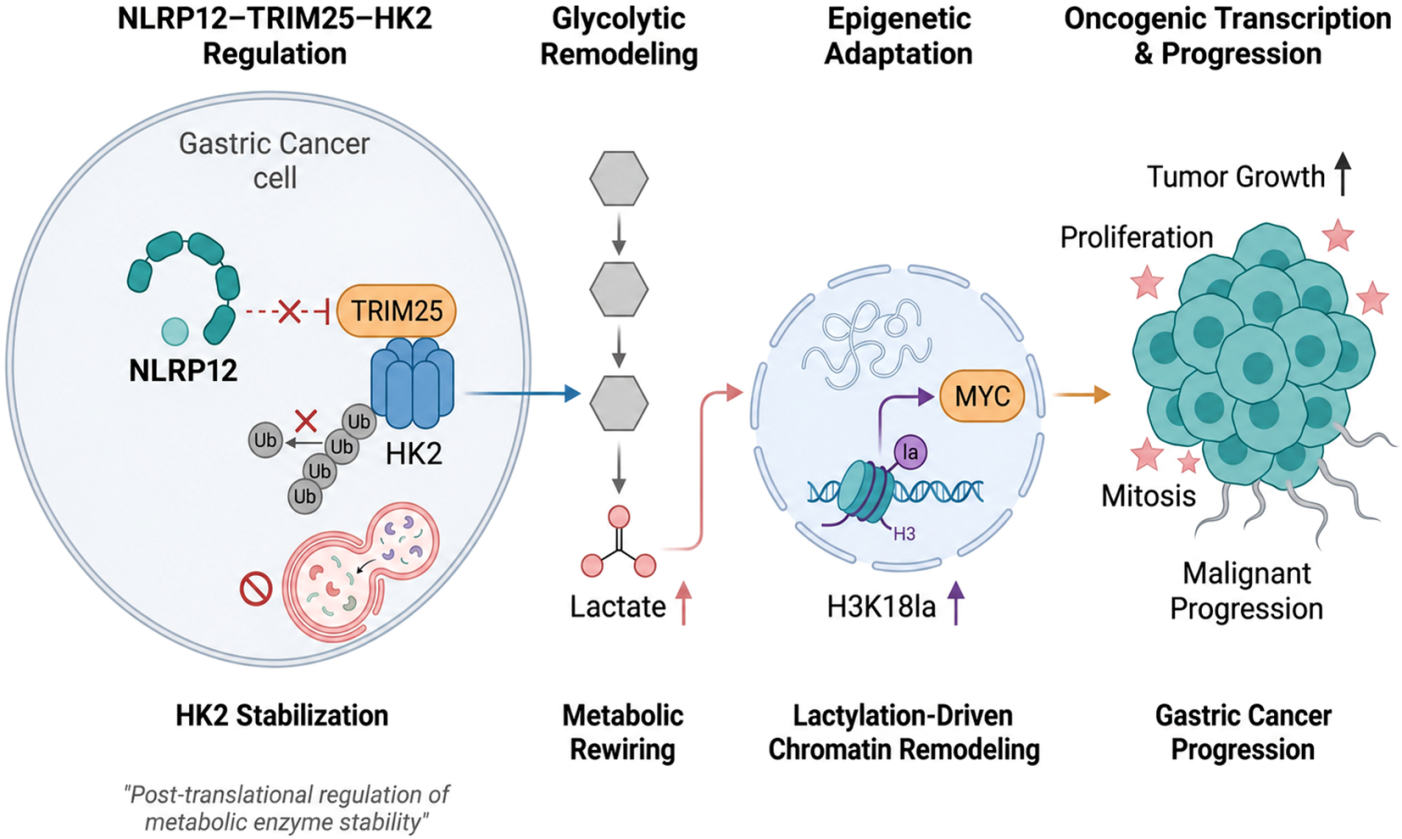
NLRP12 promotes glycolytic remodeling and lactylation-driven transcriptional output in gastric cancer. By reducing TRIM25-mediated HK2 degradation, NLRP12 stabilizes HK2, enhances glycolysis and lactate production, increases histone H3K18 lactylation, and reinforces MYC-related transcriptional programs, thereby promoting gastric cancer progression.

A particularly important aspect of this study is that it linked NLRP12-mediated metabolic rewiring to epigenetic adaptation. Increased lactate production downstream of HK2 stabilization was associated with elevated histone H3K18 lactylation, which in turn reinforced oncogenic transcriptional programs including MYC-related output (54, 55). Thus, in gastric cancer, NLRP12 appears to function not as an anti-inflammatory brake but as a metabolic facilitator that connects enzyme stability, glycolytic adaptation, and chromatin-level regulation. This mechanism is conceptually significant because it places NLRP12 within the emerging axis of metabolism-dependent epigenetic remodeling in cancer. It also represents one of the clearest examples in which NLRP12 is directly integrated into a tumor-promoting biochemical circuit rather than merely associated with disease severity. This gastric cancer study has broader implications for how the field interprets NLRP12 biology. First, it demonstrates that NLRP12 can be embedded in post-translational protein quality-control pathways, rather than acting only through classical inflammatory transcriptional signaling. Second, it suggests that NLRP12 may become tumor-promoting in contexts where metabolic plasticity and lactate-driven adaptation are dominant determinants of tumor fitness. Finally, it raises the possibility that some of the context dependency of NLRP12 arises from whether the surrounding tumor ecosystem is primarily constrained by inflammatory injury or by metabolic competition and anabolic demand. In such settings, NLRP12 may be repurposed from a restraining factor into a facilitator of malignant adaptation. **Figure 6** illustrates how NLRP12 functions as a metabolic facilitator in gastric cancer by coupling HK2 stabilization to glycolytic remodeling, lactate-dependent histone lactylation, and oncogenic transcriptional output.

### 4.2 NLRP12 is linked to poor prognosis and immune-associated aggressiveness in ovarian cancer

The evidence supporting a tumor-promoting role of NLRP12 in ovarian cancer remains less mechanistically established than that in gastric cancer. Current findings are mainly derived from integrative bioinformatic analyses, prognostic associations, immune-infiltration correlations, and limited functional validation. Therefore, NLRP12 should currently be interpreted as an adverse immune-associated biomarker and candidate functional contributor in ovarian cancer, rather than as a fully defined oncogenic driver. Nevertheless, the available data are remarkably consistent in suggesting that elevated NLRP12 expression is associated with poor prognosis and an immune-related aggressive phenotype. In epithelial ovarian cancer, increased NLRP12 expression was reported to correlate significantly with poor survival and immune infiltration (56). The study also suggested associations with immune-related pathways and indicated that NLRP12 may mark a biologically active, microenvironmentally engaged disease state rather than a passive expression change.

**Figure 7:**
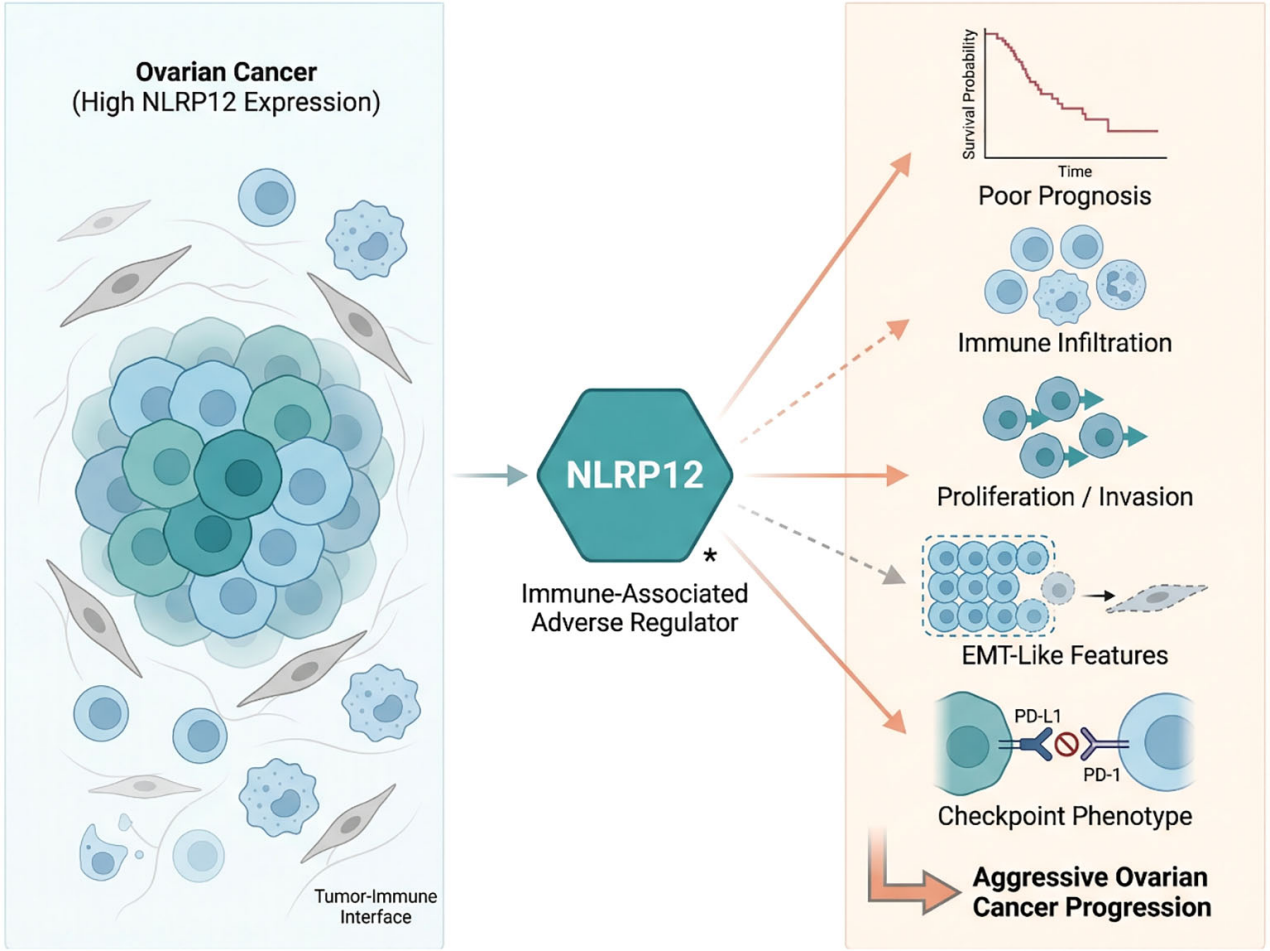
NLRP12 is linked to poor prognosis and immune-associated aggressiveness in ovarian cancer. Elevated NLRP12 expression is associated with immune infiltration, proliferation, invasion, EMT-like behavior, and immune checkpoint-related phenotypes, supporting its role as an immune-associated adverse biomarker with emerging functional relevance in ovarian cancer progression.

A subsequent 2025 study further strengthened this pattern by showing that NLRP12 is upregulated in ovarian cancer and that high expression correlates with negative prognosis and increased immune-cell infiltration. *In vitro* assays in that study suggested that NLRP12 promotes ovarian cancer cell proliferation and invasion, while additional analyses linked NLRP12 to epithelial–mesenchymal transition-related behavior and immune checkpoint-associated phenotypes. These findings do not yet establish a definitive lineage-resolved mechanism comparable to the STK38/GSK3β/β-catenin axis in colorectal cancer or the TRIM25/HK2 pathway in gastric cancer. However, they do support the view that NLRP12 may act as a functionally relevant biomarker and potential driver of aggressive progression in ovarian cancer. What is especially interesting in the ovarian cancer literature is the convergence of prognostic, immune, and functional signals. Rather than indicating a purely tumor cell-autonomous proliferative role, these studies suggest that NLRP12 may sit at the interface of malignant phenotype and immune-context remodeling. This is important because ovarian cancer progression is strongly influenced by stromal and immune composition, and genes associated with immune infiltrates often reflect a broader ecological state of the tumor. Accordingly, NLRP12 in ovarian cancer is best viewed at present as an immune-associated adverse biomarker with preliminary functional evidence, rather than a mechanistically validated oncogenic regulator. More refined studies using single-cell profiling, spatial analysis, and lineage-specific perturbation will be required to determine whether its dominant function arises from tumor cells, myeloid cells, stromal compartments, or reciprocal interactions among these populations. More refined work will be needed to determine whether its dominant action arises in tumor cells, myeloid cells, or through reciprocal signaling between both compartments. **Figure 7** summarizes the current evidence linking elevated NLRP12 expression to poor prognosis, immune-associated remodeling, and aggressive phenotypes in ovarian cancer, while highlighting that its dominant cellular source and precise mechanism remain incompletely defined.

### 4.3 High NLRP12 expression is associated with malignant progression in glioma

Evidence from glioma also suggests a possible tumor-promoting association of NLRP12, although the current support is largely based on clinical correlation, expression analysis, and limited *in vitro* functional experiments. Therefore, the role of NLRP12 in glioma should be considered hypothesis-generating rather than mechanistically established (57). In a 2023 study, high NLRP12 expression was associated with tumor grade and poor prognosis, and the authors concluded that NLRP12 may serve as an independent prognostic factor in glioma. Functional experiments further showed that siRNA-mediated downregulation of NLRP12 inhibited glioma cell proliferation, migration, and invasion, while also reducing expression of malignancy-associated proteins such as VEGF, N-cadherin, and cyclin D1 (58–61). These findings suggest that NLRP12 may be functionally associated with aggressive glioma behavior; however, the upstream regulators, downstream effectors, and cellular compartment responsible for this association remain to be defined.

Even so, the glioma literature should be interpreted carefully. The study provides clinically relevant evidence and a useful functional starting point, but it does not yet fully define the upstream drivers or downstream molecular partners that make NLRP12 oncogenic in the central nervous system tumor context. It remains unclear whether NLRP12 acts primarily through glioma cell-intrinsic pathways, through interactions with microglial or macrophage compartments, or through adaptation to the hypoxic and inflammatory niche characteristic of aggressive gliomas. Nevertheless, the available data are consistent with the broader idea that NLRP12 can support malignant progression in tumor types where inflammatory and developmental signaling are tightly intertwined.

### 4.4 Reconciling the paradox: why can NLRP12 switch from tumor-suppressive to tumor-promoting?

When reconciling the divergent roles of NLRP12 across cancers, it is important to distinguish mechanistically established findings from associative or hypothesis-generating observations. The gastric cancer HK2–glycolysis–H3K18la axis and the colorectal cancer STK38/GSK3β/β-catenin axis are supported by relatively direct mechanistic evidence, whereas the tumor-promoting roles proposed in ovarian cancer and glioma currently rely more heavily on bioinformatic correlations and limited functional validation (11). At first glance, the divergent literature on NLRP12 appears contradictory. In some tumors, NLRP12 limits noncanonical NF-κB, JNK, or Wnt/β-catenin signaling and suppresses tumorigenesis; in others, it correlates with poor prognosis or directly supports glycolysis, invasive behavior, and immune-associated progression. However, these findings become more coherent when interpreted through a context-dependent framework. One explanation is that the dominant biological function of NLRP12 depends on which signaling constraint matters most in a given tissue. In inflammation-associated epithelial tumors such as colitis-associated colorectal cancer, NLRP12 serves primarily as a brake on chronic inflammatory amplification and oncogenic transformation. In contrast, in metabolically adaptive tumors such as gastric cancer, NLRP12 may be recruited into protein-stability and glycolytic circuits that enhance tumor fitness rather than suppress it (62, 63). In ovarian cancer and glioma, where immune composition and microenvironmental plasticity strongly influence disease behavior, NLRP12 may reflect or reinforce an aggressive tumor ecosystem rather than simply acting through one linear pathway. A second explanation is that the biological output of NLRP12 is likely shaped by cellular compartment. Tumor-cell-intrinsic NLRP12 may suppress certain oncogenic pathways in one setting, whereas immune-cell-associated NLRP12 may promote immunosuppressive remodeling in another. This distinction becomes especially relevant when interpreting clinical expression studies, because bulk-tumor measurements cannot readily distinguish whether high NLRP12 originates from malignant cells, stromal cells, infiltrating myeloid cells, or combinations thereof. The same expression pattern may therefore carry fundamentally different biological meanings across cancer types.

Taken together, these observations argue against a binary model of NLRP12 function. Instead, NLRP12 should be viewed as a conditionally wired signaling regulator whose impact depends on lineage-specific circuitry, metabolic state, and tumor ecosystem composition. This interpretation not only reconciles the apparent paradox in the current literature, but also sets up the next section of this review: the role of NLRP12 in the tumor immune microenvironment, where its context dependency becomes even more evident. To provide an integrated overview of the heterogeneous roles of NLRP12 across malignancies, the expression patterns, functional effects, and representative mechanisms reported in different cancer types are summarized in **Table 1**.

**Table 1:** Expression patterns, functional roles, and mechanisms of NLRP12 across cancer types.

Cancer type	NLRP12 expression pattern	Predominant role	Main mechanism/pathway	Major phenotypic effect	Representative evidence
Colitis-associated colorectal cancer	Functional loss is associated with worse disease	Tumor suppressor	Negative regulation of noncanonical NF-κB via NIK/TRAF3/p100-p52	Suppresses colitis, inflammation-driven tumorigenesis, and tumor-promoting inflammatory mediators	Allen et al., *Immunity*, 2012 (30)
Colorectal cancer	NLRP12 deficiency enhances tumor signaling	Tumor suppressor	STK38/GSK3β/β-catenin axis	Reduces proliferation, EMT-like changes, matrix remodeling, and invasive progression	Khan et al., *J Clin Invest*, 2023 (38)
Hepatocellular carcinoma	NLRP12 loss promotes tumorigenesis	Tumor suppressor	Restraint of JNK/cJun/cMyc signaling; preservation of p21	Suppresses hepatocyte inflammation, proliferation, and HCC development	Udden et al., *eLife*, 2019 (24)
Triple-negative breast cancer	Reduced expression in tumor context	Tumor suppressor	Inhibition of canonical NF-κB signaling	Suppresses proliferation, migration, invasion, and xenograft growth	Kuang et al., *Cell Biochem Biophys*, 2023 (34)
Gastric cancer	High/functional NLRP12 promotes progression	Tumor promoter	TRIM25–HK2 regulation, enhanced glycolysis, lactate production, H3K18la, MYC output	Promotes glycolytic remodeling and tumor growth	Zhou et al., *Cell Death Dis*, 2025 (11)
Epithelial ovarian cancer	Increased expression associated with poor outcome	Tumor promoter/adverse biomarker	Immune infiltration-related programs; possible EMT-related functions	Associated with poor prognosis, immune infiltration, proliferation, and invasion	Ma et al., *J Gene Med*, 2024 (56); Xie et al., *Mol Carcinog*, 2025 (92)
Intracranial glioma	High expression associated with high grade and poor prognosis	Tumor promoter	Mechanism incompletely defined; associated with VEGF, N-cadherin, cyclin D1	Promotes proliferation, migration, and invasion	Cheng et al., *J Chin Med Assoc*, 2023 (57)
Lung adenocarcinoma TAMs	High expression in TAMs	Tumor promoter in microenvironment	NLRP12/C1qA positive feedback via LILRB4/NF-κB	Drives protumor macrophage polarization, suppresses T-cell responses, promotes immunosuppression	Yin et al., *Cancer Immunol Immunother*, 2024 (64)
Prostate cancer	Increased expression in malignant tissue	Putative inflammasome-related role	Inflammasome-associated cytokine regulation	Suggests contribution to tumor-associated inflammation	Karan et al., *Sci Rep*, 2017 (85)
PANoptosis-related context	Stimulus-dependent	Stress-response regulator			

## 5 NLRP12 in the tumor immune microenvironment

It is important to distinguish the immune or stromal functions of NLRP12 from its tumor-cell-intrinsic activities discussed above. In tumor cells, NLRP12 may directly regulate oncogenic signaling, proliferation, invasion, or metabolic adaptation. By contrast, in stromal and immune compartments, NLRP12 primarily affects tumor progression indirectly by reshaping macrophage polarization, inflammatory signaling, immune infiltration, and T-cell-mediated antitumor responses. This compartment-specific distinction is essential for interpreting why NLRP12 expression may correlate with opposite biological outcomes across different cancers. The role of NLRP12 in cancer cannot be understood solely from tumor cell-intrinsic signaling. Increasing evidence indicates that NLRP12 also participates in shaping the immune composition and functional state of the tumor microenvironment (TME), particularly through myeloid-lineage cells. This dimension is especially important because immune context often determines whether inflammatory signaling restrains tumor growth or instead becomes rewired into immunosuppressive, tumor-supportive circuitry. In this regard, NLRP12 appears capable of influencing macrophage polarization, immune infiltration patterns, and antitumor T-cell activity, thereby functioning as a regulator of tumor ecology rather than only a modulator of intracellular signaling.

### 5.1 NLRP12 shapes tumor-associated macrophage polarization and immunosuppressive signaling

The clearest mechanistic evidence for an immune microenvironmental role of NLRP12 comes from lung adenocarcinoma, where NLRP12 was found to be predominantly expressed in tumor-associated macrophages (TAMs) and linked to poor patient prognosis. In that study, NLRP12 formed a positive-feedback loop with C1qA and promoted protumor macrophage polarization through the LILRB4/NF-κB pathway (64). Functionally, NLRP12-overexpressing TAMs enhanced malignant progression of tumor cells while suppressing T-cell proliferation and cytotoxic function. Conversely, genetic loss of NLRP12 reversed macrophage protumor polarization, enhanced T-cell antitumor immunity, and suppressed tumor growth (65, 66). These findings are particularly significant because they show that NLRP12 can promote cancer not by directly enhancing tumor cell proliferation, but by enforcing an immunosuppressive macrophage state that protects the tumor ecosystem. Thus, the LUAD model provides a clear example of microenvironmental NLRP12 function, in which NLRP12 acts through tumor-associated macrophages rather than through a tumor-cell-autonomous mechanism.

**Figure 8:**
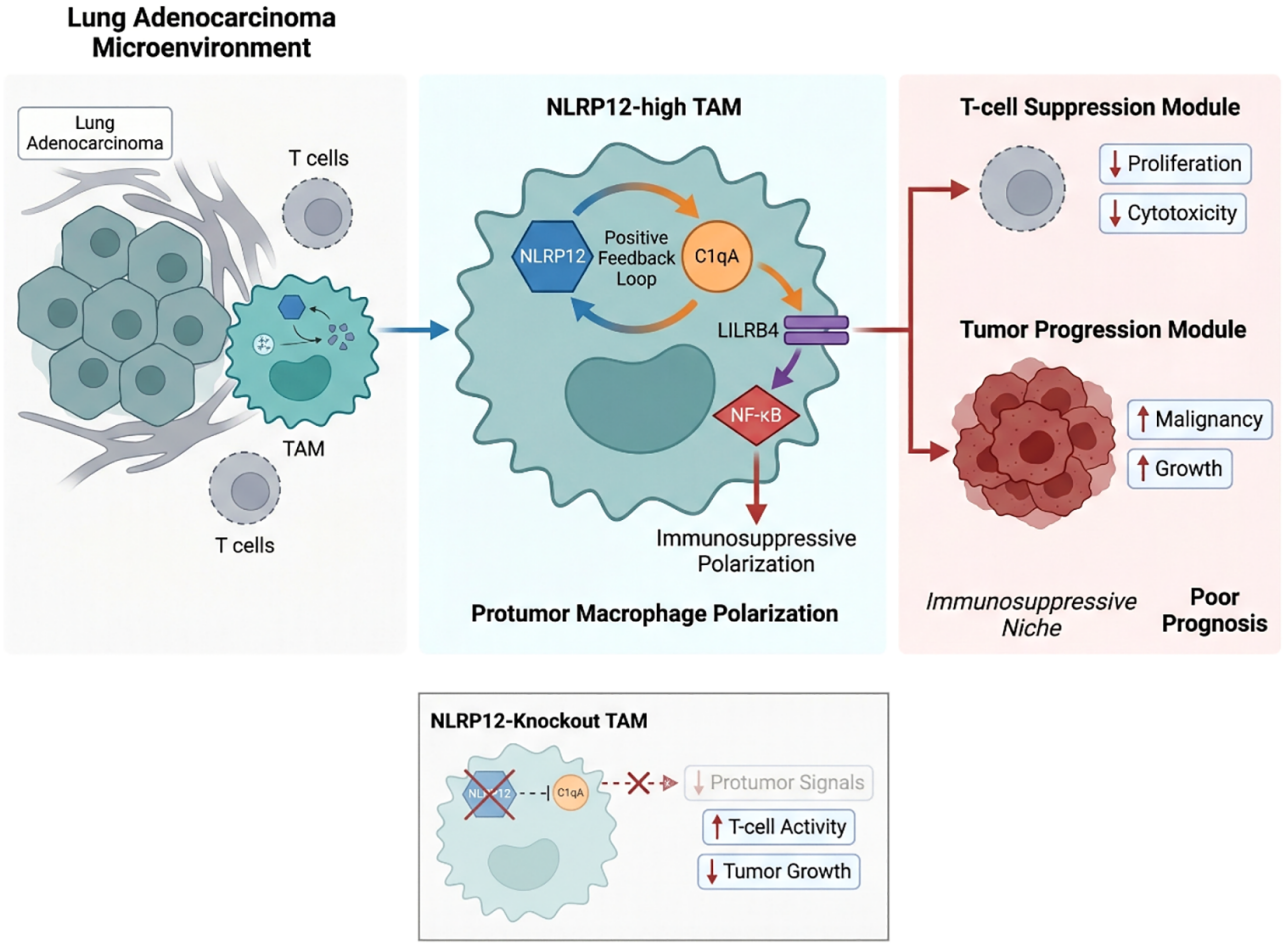
NLRP12 shapes tumor-associated macrophage polarization and immunosuppressive signaling in lung adenocarcinoma. Predominantly expressed in TAMs, NLRP12 forms a positive-feedback loop with C1qA and activates the LILRB4/NF-κB pathway, thereby promoting protumor macrophage polarization, suppressing T-cell proliferation and cytotoxic function, and enhancing tumor progression.

This TAM-centered mechanism also helps explain why bulk-tumor NLRP12 expression may sometimes associate with adverse outcomes even when tumor-cell-intrinsic studies suggest suppressive functions in other cancers. If a substantial fraction of NLRP12 signal arises from macrophages or other myeloid cells, then high expression may reflect an immune-suppressive niche rather than a protective epithelial checkpoint. The LUAD study therefore provides a crucial conceptual correction for the field: the biological meaning of NLRP12 cannot be inferred from expression level alone without considering cellular origin. This is likely to be highly relevant not only for lung adenocarcinoma but also for other tumors rich in myeloid infiltration (**Figure 8**).

### 5.2 NLRP12 is associated with immune infiltration across tumor types

Beyond the macrophage-focused evidence in LUAD, ovarian cancer studies have also suggested that NLRP12 is closely linked to immune infiltration. In epithelial ovarian cancer, elevated NLRP12 expression was associated with poor prognosis and correlated with multiple immune infiltrates. A later integrative study similarly identified NLRP12 as an immune-related adverse biomarker in ovarian cancer and linked it to immune-cell infiltration and immune checkpoint-associated phenotypes. Although these studies do not yet establish a definitive mechanism, they consistently place NLRP12 within an immune-active tumor context and support the idea that its prognostic value may be mediated, at least in part, through immune ecological remodeling rather than exclusively through tumor cell-autonomous effects.

At the same time, these associations should be interpreted cautiously. Most current analyses of immune infiltration rely on transcriptomic deconvolution or correlative bioinformatics frameworks, which are valuable for hypothesis generation but cannot by themselves define causality. High NLRP12 expression may indicate active participation in immune remodeling, but it may also serve as a surrogate marker of broader inflammatory states, myeloid enrichment, or stromal complexity (67–71). Accordingly, future work will need to combine single-cell profiling, spatial transcriptomics, and lineage-specific perturbation models to determine whether NLRP12 actively drives immune recruitment and polarization or instead marks tumors that are already immunologically reprogrammed.

### 5.3 NLRP12 and immune escape: direct effector or contextual amplifier?

A central unresolved question is whether NLRP12 should be considered a direct mediator of immune escape or a contextual amplifier of pre-existing immunosuppressive programs. The available evidence currently favors the latter, more nuanced view. In lung adenocarcinoma, NLRP12 does not appear to function as a classical immune checkpoint molecule analogous to PD-L1; instead, it operates within a myeloid signaling circuit that amplifies protumor macrophage polarization and weakens T-cell function (72, 73). In ovarian cancer, NLRP12 correlates with immune infiltration and immune checkpoint-associated patterns, but the causal direction remains unclear. Together, these data suggest that NLRP12 may be best understood as a regulator of the immune conditions that enable escape, rather than as a single linear checkpoint effector. This distinction matters therapeutically. If NLRP12 primarily amplifies immunosuppressive myeloid ecosystems, then targeting NLRP12 or its downstream partners may be most effective in tumors where macrophage-driven immune dysfunction is dominant. In that case, NLRP12-centered interventions might complement T-cell-directed immunotherapies by altering the upstream ecological constraints that blunt cytotoxic immunity. Conversely, in tumors where NLRP12 acts mainly within malignant cells as a suppressor of oncogenic signaling, indiscriminate inhibition could be counterproductive. Thus, the immune microenvironmental role of NLRP12 further reinforces the central thesis of this review: its biological significance in cancer is fundamentally context dependent and must be interpreted in a cell-resolved manner.

### 5.4 Implications for tumor ecosystem biology

Taken together, current evidence indicates that NLRP12 should be incorporated into a broader tumor ecosystem framework. Rather than functioning solely as an intracellular inflammatory sensor, NLRP12 can influence how immune cells interpret and reinforce local signals within the TME. In macrophage-rich tumors, it may support complement-linked, NF-κB-dependent immunosuppressive loops (74–76). In immune-infiltrated ovarian tumors, it may reflect a convergence point between adverse prognosis and immune remodeling. These observations extend the relevance of NLRP12 beyond tumor initiation or proliferation and place it within the dynamic network of tumor–immune reciprocity that shapes progression and therapeutic responsiveness. From a conceptual standpoint, this immune-centered perspective also helps reconcile some of the apparent contradictions in the literature. NLRP12 may suppress tumorigenesis when acting within epithelial or parenchymal cells to restrain oncogenic and inflammatory signaling, yet promote tumor progression when engaged in macrophage-driven immunosuppressive circuits. The biological output therefore depends not simply on whether NLRP12 is present, but on where it is expressed and which cellular network it is embedded in. This cell-context-aware interpretation is likely essential for any future effort to translate NLRP12 into a clinically meaningful biomarker or therapeutic target.

## 6 NLRP12 and cancer metabolism

Cancer metabolism has traditionally been discussed through the lens of oncogenic drivers, nutrient competition, and microenvironmental adaptation. However, increasing evidence suggests that inflammatory regulators can also participate directly in metabolic rewiring, thereby influencing tumor growth, stress tolerance, and therapeutic plasticity. In this context, NLRP12 is beginning to emerge as more than an innate immune regulator. While the current literature remains limited, available studies indicate that NLRP12 can modulate glycolytic flux, lactate-associated chromatin remodeling, and potentially broader interfaces between inflammatory signaling and metabolic adaptation. The strongest mechanistic evidence comes from gastric cancer, where NLRP12 was shown to stabilize HK2 and drive a glycolysis–lactylation axis that promotes tumor progression.

### 6.1 From inflammatory regulator to metabolic signaling node

One of the most important conceptual shifts in recent NLRP12 research is the recognition that its biological role is not confined to NF-κB regulation or inflammasome-associated signaling. The gastric cancer study demonstrates that NLRP12 can directly control the stability of a core metabolic enzyme, thereby influencing cellular bioenergetics and biosynthetic adaptation. This expands the functional identity of NLRP12 from an inflammation-modifying molecule to a signaling node that can support tumor metabolism under selected conditions. Rather than merely shaping cytokine output or inflammatory tone, NLRP12 may in some contexts regulate the biochemical infrastructure that sustains malignant growth. This shift is highly relevant to cancer biology because metabolic rewiring is not a secondary byproduct of transformation; it is a central determinant of tumor fitness. Glycolysis, lactate accumulation, and downstream metabolite-sensitive chromatin changes contribute not only to ATP production and biosynthesis, but also to invasion, immune evasion, and resistance to stress (77). By entering this regulatory landscape, NLRP12 becomes part of a broader network linking inflammation, metabolism, and tumor adaptation. This also helps explain why the biological consequences of NLRP12 can diverge sharply across tumor types: in some settings, its dominant effect may be anti-inflammatory restraint, whereas in others it may enhance metabolic plasticity.

### 6.2 NLRP12 promotes the HK2–glycolysis–lactate–H3K18la axis in gastric cancer

The clearest evidence for a metabolic role of NLRP12 comes from gastric cancer, where NLRP12 stabilizes hexokinase 2 (HK2) and promotes glycolysis, leading to lactate production and associated histone H3K18 lactylation (11). Emerging evidence suggests that similar mechanisms may also operate in other cancers where glycolytic remodeling contributes to immune evasion, including hepatocellular carcinoma, colorectal cancer, and triple-negative breast cancer (63, 78, 79). Although direct mechanistic validation outside gastric cancer is limited, these observations indicate that NLRP12-mediated metabolic reprogramming could broadly influence tumor-promoting, immunosuppressive microenvironments across diverse malignancies. Future studies should evaluate whether NLRP12 similarly modulates glycolysis and immune suppression in additional tumor types, including both tumor cells and tumor-associated stromal or immune compartments, to determine the generality of this mechanism and its potential therapeutic implications.

The consequences of this metabolic effect extended beyond glycolytic flux itself. Increased lactate production in NLRP12-high gastric cancer cells was associated with elevated histone H3K18 lactylation, linking NLRP12 activity to metabolite-dependent epigenetic remodeling. The same study further connected this chromatin change to increased MYC transcriptional output, thereby establishing a mechanistic chain from NLRP12 to enzyme stability, glycolysis, lactate accumulation, histone modification, and oncogenic transcription. In conceptual terms, this places NLRP12 within an integrated metabolic–epigenetic circuit rather than a purely inflammatory one. This pathway is notable for several reasons. First, it shows that NLRP12 can support tumor progression without acting through canonical inflammatory cascades. Second, it suggests that the pro-tumor role of NLRP12 in some cancers may depend on its ability to reinforce metabolic adaptation rather than simply altering cytokine signaling. Third, it raises the possibility that NLRP12 could contribute to broader lactate-centered tumor biology, including chromatin plasticity, phenotypic switching, and immune modulation, although direct evidence for these downstream extensions remains limited at present. Accordingly, the HK2–lactate–H3K18la axis should currently be viewed as the most substantiated metabolic mechanism for NLRP12 in cancer.

### 6.3 Potential links between NLRP12 and broader metabolic adaptation

Although direct mechanistic evidence outside gastric cancer remains sparse, the available literature suggests several plausible ways in which NLRP12 may intersect with broader metabolic adaptation. First, its established role in inflammatory signal control places it near pathways that influence nutrient allocation, redox balance, and mitochondrial stress responses. Second, its context-dependent effects in tumors with strong metabolic and inflammatory coupling imply that NLRP12 may help determine how cells respond to nutrient stress or lactate-rich microenvironments. Third, its capacity to regulate pathway-specific tumor behavior in colorectal cancer and hepatocellular carcinoma suggests that metabolic effects may coexist with, or be secondary to, signaling effects in some settings. These possibilities remain inferential rather than proven, but they provide a rationale for expanding future studies beyond the currently dominant inflammatory framework.

From a tumor ecosystem perspective, the metabolic role of NLRP12 may also intersect with immune remodeling. Lactate accumulation is well known to alter myeloid-cell polarization, T-cell dysfunction, and stromal behavior, and therefore NLRP12-driven enhancement of glycolysis could theoretically reinforce an immunosuppressive TME (78–81). This idea is attractive because it could unify the tumor-promoting metabolic role observed in gastric cancer with the immunosuppressive functions seen in macrophage-rich tumors. However, direct experimental linkage between NLRP12-driven tumor metabolism and immune suppression has not yet been firmly established and should not be overstated. At present, the most defensible conclusion is that NLRP12 can regulate cancer metabolism directly in at least one tumor type, and that this role may have wider consequences for tumor ecosystem behavior.

### 6.4 Translational implications of NLRP12-dependent metabolic rewiring

The emerging metabolic role of NLRP12 has important translational implications. If NLRP12 promotes tumor fitness by stabilizing HK2 and reinforcing lactate-dependent chromatin remodeling, then tumors with high NLRP12 expression may display heightened dependence on glycolytic adaptation. In principle, this could create therapeutic opportunities for combining NLRP12-centered stratification with metabolic inhibitors targeting glycolysis, lactate production, or downstream epigenetic consequences of lactate accumulation (82–84). At the same time, the context-dependent nature of NLRP12 cautions against generalized targeting. In cancers where NLRP12 acts primarily as a suppressor of Wnt/β-catenin, JNK, or NF-κB signaling, direct inhibition could be harmful rather than beneficial.

Taken together, current evidence supports a revised view in which NLRP12 is not only an inflammatory regulator but also an emerging participant in cancer metabolic adaptation. The gastric cancer literature provides the first robust mechanistic foundation for this concept, while studies in other tumors suggest that the metabolic dimension of NLRP12 may be broader than currently appreciated. Defining when NLRP12 acts as a metabolic facilitator, how this intersects with immune ecology, and whether these effects are tumor-cell-intrinsic or microenvironmentally reinforced will be essential questions for future research.

## 7 NLRP12, PANoptosis-related signaling, and cancer-associated stress adaptation

Although much of the recent cancer-related literature on NLRP12 has focused on inflammatory signaling, oncogenic pathway modulation, immune remodeling, and metabolism, the molecule cannot be fully understood without considering its relationship to inflammasome-associated biology and regulated inflammatory cell death. Historically, NLRP12 was classified within the NLRP family because of its structural similarity to inflammasome-forming sensors, and early cancer-related studies explored its potential contribution to inflammasome signaling in tumor contexts such as prostate cancer (85). More recently, the field has broadened substantially with the recognition that NLRP12 can also participate in PANoptosis-related signaling, placing it within a wider framework of stress-induced cell death integration. Together, these developments suggest that NLRP12 may influence cancer not only by modulating growth and immunity, but also by shaping how cells respond to inflammatory injury, death-inducing stress, and damage-associated signaling. However, it should be emphasized that direct cancer-specific validation of NLRP12-mediated PANoptosis remains limited. Most current evidence linking NLRP12 to PANoptotic signaling comes from inflammatory or infection-related models rather than tumor-focused systems. Therefore, in the cancer context, NLRP12-mediated PANoptosis should currently be interpreted as an emerging mechanistic possibility rather than an established tumor-regulatory pathway.

### 7.1 Classical inflammasome-related functions of NLRP12

Early work linked NLRP12 to inflammasome biology and suggested that its expression may be associated with inflammatory cytokine regulation in cancer. In prostate cancer, Karan and colleagues analyzed several inflammasome sensors and reported increased NLRP12 expression in malignant tissue compared with adjacent benign tissue, together with evidence that inflammasome-related proteins such as ASC, pro-caspase-1, IL-1β, and IL-18 were enriched in aggressive prostate cancer cells (86, 87). The authors interpreted these findings as support for a role of the NLRP12 inflammasome complex in regulating inflammatory cytokines in prostate cancer. While this study did not establish a deeply resolved mechanistic pathway comparable to later work in colorectal or gastric cancer, it remains important because it introduced NLRP12 into the cancer field as more than a passive immune marker and suggested that inflammasome-related outputs could contribute to tumor-associated inflammation. At the same time, subsequent literature has made it clear that NLRP12 should not be viewed as a conventional inflammasome sensor in the same way as NLRP3. Its inflammasome-associated activity appears more selective, context-dependent, and integrated with other signaling functions. Recent reviews now describe NLRP12 as a molecule that can participate in both inflammasome and PANoptosome biology depending on stimulus context, rather than acting through a single fixed death-signaling program. This shift is important for cancer interpretation because it implies that NLRP12-related inflammatory outputs may vary substantially according to cell type, trigger, and signaling environment.

### 7.2 HCK-regulated NLRP12-mediated PANoptosis

A major recent advance came from the 2025 PNAS study showing that HCK regulates NLRP12-mediated PANoptosis. In that work, the authors identified hematopoietic cell kinase (HCK) as a regulator of the NLRP12-dependent PANoptotic program and showed that HCK is induced during NLRP12-driven PANoptosis. Knockdown of HCK suppressed NLRP12-mediated PANoptosis, supporting a functional requirement for this kinase in the death pathway (88). The study also provided evidence that HCK interacts with NLRP12, with computational and experimental analyses pointing to a region between the NACHT and PYD-associated domains as important for this regulatory interaction.

This finding is significant because PANoptosis is now understood as an integrated inflammatory cell death program combining features of pyroptosis, apoptosis, and necroptosis, executed through multiprotein PANoptosome assemblies rather than isolated linear pathways. The identification of HCK as a regulator of NLRP12-mediated PANoptosis places NLRP12 within this broader death-signaling network and expands its functional identity from an inflammatory regulator to a sensor or scaffold participating in stress-responsive death coordination. In conceptual terms, this means that NLRP12 may influence not only whether inflammatory signals are generated, but also whether damaged or stressed cells proceed toward a highly inflammatory form of death. Although this work was not performed in a tumor model, it provides a mechanistic basis for considering whether NLRP12-dependent PANoptotic signaling may operate under cancer-associated stress conditions. Nevertheless, its relevance to tumor biology remains hypothetical until validated in cancer-specific models, such as tumor cells, tumor-bearing mice, or immune cells isolated from the tumor microenvironment. PANoptosis is increasingly viewed as an important determinant of tumor cell elimination, inflammatory remodeling, and treatment response, and recent reviews emphasize that PANoptotic pathways may influence cancer progression, drug resistance, and tumor–immune interactions. From that perspective, NLRP12-mediated PANoptosis may eventually prove relevant to how cancer cells or tumor-associated immune cells respond to inflammatory therapies, cytotoxic stress, or microenvironmental injury. At present, however, direct cancer-specific evidence remains limited, and this translational link should be presented as a promising direction rather than an established conclusion.

### 7.3 NLRP12, inflammatory cell death, and stress adaptation in cancer

At present, direct evidence connecting NLRP12-mediated PANoptosis to cancer progression, metastasis, or therapy response remains sparse. Therefore, the discussion below should be viewed as a cancer-oriented extrapolation from PANoptosis biology rather than a fully established tumor-specific mechanism. The relationship between NLRP12 and cancer-associated stress adaptation is likely to be multifaceted. In one direction, NLRP12 may limit tumorigenesis by buffering inflammatory signaling and thereby preventing chronic tissue injury from escalating into proliferative or oncogenic reprogramming, as seen in colitis-associated cancer and hepatocellular carcinoma. In another direction, its emerging role in PANoptosis suggests that NLRP12 may help determine whether inflammatory stress culminates in cell survival, nonlethal adaptation, or immunogenic cell death. These two functions are not mutually exclusive: a molecule that restrains chronic inflammatory signaling in one context may still promote death execution under a different trigger landscape. This duality is consistent with the broader theme of NLRP12 biology, namely that its output depends on stimulus context, cellular compartment, and network integration. From a cancer standpoint, this raises several possibilities. NLRP12-dependent death signaling could affect the survival of malignant cells exposed to inflammatory or therapeutic stress. It could also shape the behavior of myeloid or stromal compartments by altering cytokine release and the immunogenic consequences of dying cells. In tumors characterized by persistent inflammatory pressure, NLRP12 may therefore function at a crossroads between stress tolerance and inflammatory cell death. However, direct evidence connecting NLRP12-mediated PANoptosis to tumor control, metastasis, or treatment sensitivity is still sparse, and further mechanistic studies will be needed to determine which cell populations are most affected and under what therapeutic conditions these pathways become relevant.

### 7.4 Therapeutic implications of NLRP12-linked cell death regulation

The emerging connection between NLRP12 and PANoptosis has potentially important translational implications (89–91). In principle, if NLRP12-dependent PANoptotic signaling can be activated in tumor cells, it may provide a route to overcome resistance mechanisms that allow escape from single-mode cell death programs. Conversely, if NLRP12 primarily supports inflammatory survival programs or pathologic inflammation in certain nonmalignant compartments, then modulating its activity could help reshape the tumor ecosystem in a more favorable direction. The challenge, once again, is context dependency. A strategy designed to enhance NLRP12-linked death signaling in one tumor type could be inappropriate in another setting where NLRP12 acts predominantly as a suppressor of oncogenic signaling or as a driver of macrophage-mediated immune suppression.

Overall, current evidence supports a broadened view in which NLRP12 should be considered part of a larger stress-response and inflammatory cell death landscape. Its earlier association with inflammasome signaling provided the initial framework, but recent PANoptosis-related findings now suggest that NLRP12 may also influence how cells integrate inflammatory, apoptotic, and necroptotic cues under pathological stress. For cancer biology, this dimension remains underdeveloped but highly promising, especially in the context of immunogenic cell death, therapy resistance, and tumor–immune reciprocity. However, these therapeutic implications remain speculative. Future studies should directly test whether NLRP12 participates in PANoptosis in tumor cells or tumor-associated immune cells, and whether manipulating this pathway alters tumor growth, immune activation, or sensitivity to chemotherapy, radiotherapy, or immunotherapy.

## 8 Clinical relevance of NLRP12 in cancer

It should be noted that most current evidence supporting NLRP12 as a prognostic or predictive marker derives from retrospective analyses of public databases or historical patient cohorts. Prospective clinical validation in well-defined patient populations is still lacking and is necessary to firmly establish its clinical utility. Future studies should focus on longitudinal monitoring of NLRP12 expression and correlation with treatment response and survival outcomes to strengthen its translational relevance. From a translational perspective, the most immediate question is not whether NLRP12 is mechanistically interesting, but whether it can serve as a clinically useful biomarker or therapeutic reference point. Current evidence suggests that NLRP12 has potential relevance in both respects, but its value is unlikely to be universal across cancers. Instead, available studies indicate that the clinical meaning of NLRP12 depends strongly on tumor type, cellular source, and the biological program with which it is associated, including inflammatory restraint, metabolic adaptation, or immune suppression. This context dependency creates both opportunity and difficulty: NLRP12 may offer prognostic and biological stratification value, yet its interpretation requires more nuance than that of a conventional single-direction biomarker.

### 8.1 Prognostic significance of NLRP12 across cancer types

One of the clearest clinical patterns emerging from the literature is that NLRP12 expression is associated with patient outcome, but not in a uniform direction. In epithelial ovarian cancer, increased NLRP12 expression has been reported to correlate with poor survival and immune infiltration, supporting its potential use as an adverse prognostic marker in that setting. A subsequent ovarian cancer study similarly identified NLRP12 as a prognostically relevant immune-related factor and linked high expression to tumor heterogeneity and aggressive behavior. In glioma, high NLRP12 expression was likewise associated with poor prognosis and was proposed as an independent prognostic factor. Together, these studies suggest that in several cancers, elevated NLRP12 marks biologically aggressive disease rather than protective inflammatory control. At the same time, outcome interpretation must be made in light of the tumor-specific functional literature. In colorectal cancer, hepatocellular carcinoma, and triple-negative breast cancer, mechanistic studies support tumor-suppressive roles for NLRP12, implying that reduced expression or loss of function may be biologically unfavorable in those contexts, even when formal large-scale prognostic datasets remain less developed than in ovarian cancer or glioma. This means that the prognostic significance of NLRP12 cannot be generalized across tumor types: high expression may indicate adverse biology in one malignancy, whereas diminished expression may reflect loss of tumor restraint in another. This bidirectional pattern is a defining feature of NLRP12 as a clinical variable.

### 8.2 NLRP12 as a biomarker of immune and metabolic states

Beyond survival association alone, NLRP12 may have value as a biomarker that reflects broader biological states within tumors. In ovarian cancer, NLRP12 expression has repeatedly been linked to immune infiltration and immune-related phenotypes, suggesting that it may capture features of an immune-remodeled tumor ecosystem rather than simply tumor burden. In lung adenocarcinoma, the observation that NLRP12 is enriched in tumor-associated macrophages and promotes immunosuppression through the C1qA/LILRB4/NF-κB axis further strengthens the idea that NLRP12 can mark a specific myeloid-dominant immune state associated with poor antitumor immunity. Under this interpretation, NLRP12 may function as an ecosystem biomarker that reflects the presence of protumor immune circuitry. The gastric cancer literature adds a complementary dimension by suggesting that NLRP12 may also reflect metabolic adaptation. Because NLRP12 promotes HK2 stability, glycolysis, lactate production, and histone H3K18 lactylation in gastric cancer, tumors with elevated NLRP12 may display a metabolic configuration characterized by heightened glycolytic dependence and lactate-linked epigenetic remodeling. Although this metabolic biomarker role has so far been defined mainly in a single tumor type, it opens the possibility that NLRP12 could be used not only for prognosis, but also for identifying tumors enriched for specific metabolic or immune states that may influence therapy response. This possibility is promising, but it remains preliminary and will require validation in larger clinical cohorts.

### 8.3 Potential predictive relevance for therapeutic response

An especially interesting question is whether NLRP12 has predictive value for treatment selection or immunotherapy response. The current evidence is still limited, but the ovarian cancer literature provides an early signal in this direction. The 2025 integrative study suggested that lower NLRP12 expression may be associated with a better response to immunotherapy, particularly CTLA4 blockade, based on immunogenomic analyses and supporting validation in experimental models (92). If confirmed, this would imply that NLRP12 might help stratify patients according to immune responsiveness, especially in tumors where its expression tracks with immunosuppressive or checkpoint-associated phenotypes. However, this remains an emerging observation rather than a clinically established biomarker application. Similarly, the macrophage-centered data in lung adenocarcinoma suggest that NLRP12-rich tumors may be less responsive to T-cell-directed strategies unless the upstream myeloid suppressive niche is also addressed. This raises the possibility that NLRP12 could serve as a marker for tumors in which macrophage-targeted or combination immune-modulating strategies are more relevant than single-agent checkpoint blockade. In contrast, in cancers where NLRP12 acts as a tumor suppressor, its loss may identify tumors driven by Wnt/β-catenin, JNK, or NF-κB hyperactivation, potentially guiding pathway-centered interventions rather than direct NLRP12 inhibition. Thus, the predictive value of NLRP12 is likely to be highly context specific and mechanistically anchored. The currently reported prognostic significance, biomarker potential, and possible predictive relevance of NLRP12 across different cancer contexts are summarized in **Table 2**.

**Table 2:** Clinical relevance and biomarker potential of NLRP12 in cancer.

Clinical dimension	Cancer context	Main observation	Potential clinical implication	Current limitation
Prognostic biomarker	Ovarian cancer	High NLRP12 correlates with poor survival and immune infiltration	May serve as an adverse prognostic biomarker and immune-state indicator	Mostly retrospective and bioinformatics-heavy; causal mechanism still incomplete
Prognostic biomarker	Glioma	High NLRP12 associates with malignant grade and poor prognosis	May help stratify aggressive glioma	Limited mechanistic depth and external validation
Immune ecosystem biomarker	Lung adenocarcinoma	NLRP12 enriched in TAMs and associated with immunosuppressive macrophage polarization	May identify myeloid-dominant immune-suppressed tumors	Cellular-source specificity required for interpretation
Metabolic-state biomarker	Gastric cancer	NLRP12 promotes HK2 stability, glycolysis, lactate accumulation, and H3K18la	May indicate glycolysis/lactylation-driven tumor adaptation	Evidence currently centered on one tumor type
Loss-of-restraint marker	Colorectal cancer	Reduced/absent NLRP12 unleashes β-catenin signaling and tumor progression	Loss of NLRP12 may mark pathway dysregulation rather than simple expression reduction	Clinical cohort-level validation remains limited
Loss-of-restraint marker	HCC	NLRP12 deficiency enhances JNK-driven inflammation and proliferation	Low NLRP12 may reflect hepatocyte-intrinsic loss of tumor restraint	Needs broader translational validation
Predictive biomarker for immunotherapy	Ovarian cancer	Lower NLRP12 may associate with better predicted immunotherapy response in integrative analysis	Could help refine immunotherapy stratification	Predictive value not yet prospectively validated
Composite biomarker component	Cross-cancer	NLRP12 reflects inflammation, metabolism, and immune context depending on tumor type	Best used in composite signatures with pathway and cell-type information	Not suitable as a universal one-direction marker

### 8.4 Major challenges for clinical translation

Despite its promise, several major obstacles currently limit the clinical translation of NLRP12. First, most available studies remain retrospective, single-center, or bioinformatics-driven, with relatively limited prospective validation. Second, bulk transcriptomic or immunohistochemical measurements often do not resolve the cellular origin of NLRP12 expression. This is a critical limitation because NLRP12 derived from tumor cells may carry very different biological meaning from NLRP12 derived from macrophages or other immune populations. Third, standardized detection methods, clinically actionable cutoffs, and validated companion assays are not yet available. These issues make it difficult to compare findings across tumor types or incorporate NLRP12 into routine clinical workflows. A further challenge is biological interpretation. Because NLRP12 can function as either a tumor suppressor or tumor promoter depending on context, there is no single direction of change that can be assumed to be clinically adverse. This sharply distinguishes NLRP12 from more straightforward biomarkers. Any practical use of NLRP12 in oncology will therefore require tumor-specific and cell-resolved interpretation, ideally integrating expression data with pathway context, immune composition, and metabolic state. In that sense, the clinical future of NLRP12 may lie less in its use as an isolated marker and more in its inclusion within composite stratification frameworks that better capture the ecological complexity of cancer.

### 8.5 A clinically oriented framework for interpreting NLRP12

Taken together, current data support a practical framework in which NLRP12 is interpreted along three axes: prognostic association, biological state representation, and therapeutic context. In some tumors, such as ovarian cancer and glioma, high NLRP12 expression may indicate poor prognosis and aggressive immune-associated disease. In macrophage-rich settings such as lung adenocarcinoma, NLRP12 may reflect an immunosuppressive myeloid niche. In other tumors, particularly those in which experimental data show tumor-suppressive activity, reduced NLRP12 may instead signify loss of protective signaling restraint. This framework helps reconcile the otherwise conflicting clinical literature and aligns with the central thesis of this review: NLRP12 is clinically relevant precisely because it is context dependent, not despite it.

## 9 Therapeutic perspectives and future directions

The therapeutic relevance of NLRP12 lies precisely in its complexity. Current evidence suggests that NLRP12 is involved in several biologically actionable processes, including inflammatory signal control, Wnt/β-catenin regulation, JNK restraint, glycolytic adaptation, macrophage-mediated immunosuppression, and PANoptosis-associated stress responses. However, these same studies also show that NLRP12 does not operate in a uniform direction across cancers. In some settings, restoring or preserving NLRP12 activity may be beneficial because it restrains tumor-promoting signaling, whereas in others, particularly those marked by glycolytic remodeling or immunosuppressive myeloid circuits, NLRP12 may represent a liability that should be inhibited indirectly or selectively. For this reason, the key translational question is no longer whether NLRP12 is “good” or “bad,” but under which biological conditions it becomes targetable in a therapeutically meaningful way.

### 9.1 Should NLRP12 be inhibited or restored?

At present, there is no single therapeutic answer that applies across tumor types. In colorectal cancer, hepatocellular carcinoma, and likely triple-negative breast cancer, the available mechanistic evidence suggests that NLRP12 functions as a protective checkpoint that restrains oncogenic or inflammatory signaling. This context-dependent biology also raises important safety concerns for NLRP12-targeted therapy. Systemic inhibition of NLRP12 could produce undesirable off-target effects in tissues where NLRP12 functions as a tumor suppressor. For example, blocking NLRP12 activity in colorectal, hepatic, or breast epithelial contexts might release control over Wnt/β-catenin, JNK, or NF-κB signaling, thereby enhancing inflammatory injury, proliferative signaling, or malignant progression. Therefore, therapeutic strategies aimed at suppressing NLRP12 should not be applied indiscriminately across cancers. In these contexts, direct inhibition of NLRP12 would be difficult to justify biologically and could even exacerbate tumor progression by releasing control over Wnt/β-catenin, JNK, or NF-κB-associated pathways. By contrast, in gastric cancer, where NLRP12 stabilizes HK2 and promotes glycolysis, lactate production, and H3K18 lactylation, suppressing NLRP12-related signaling may be more rational. Similarly, in lung adenocarcinoma, where NLRP12 in tumor-associated macrophages supports a C1qA/LILRB4/NF-κB immunosuppressive loop, inhibition of the NLRP12-centered macrophage program may have therapeutic value even if systemic NLRP12 inhibition would be undesirable in other tissues. This context-specific logic means that future therapeutic strategies should not treat NLRP12 as a universal drug target. Instead, NLRP12 should be approached as a stratification-dependent node whose manipulation depends on tumor lineage, cellular source, and dominant biological circuit. A practical translational framework may therefore require first determining whether NLRP12 is acting primarily in tumor cells, epithelial cells, hepatocytes, macrophages, or other compartments, and then deciding whether its dominant output in that setting is suppressive or promotive. This principle may seem obvious, but it is especially important for NLRP12 because the same molecule can sit on opposite sides of cancer biology depending on the surrounding ecosystem.

### 9.2 Targeting NLRP12-centered signaling circuits rather than NLRP12 alone

Given this complexity, directly drugging NLRP12 may not be the most realistic short-term strategy. NLRP12 appears to function largely through scaffold-like or adaptor-like mechanisms and protein–protein interactions, which may be difficult to target with conventional small molecules. A more feasible approach may be to modulate the downstream or collateral circuits through which NLRP12 exerts its tumor-relevant effects. In cancers where NLRP12 loss unleashes oncogenic signaling, such as Wnt/β-catenin, JNK, or NF-κB activation, pathway-directed intervention may be more appropriate than attempting to restore NLRP12 itself. Conversely, in cancers where NLRP12 supports tumor fitness, therapeutic leverage may be achieved by blocking the specific dependencies it creates. For example, the gastric cancer study points toward the HK2–glycolysis–lactate–H3K18la axis as a tractable metabolic vulnerability downstream of NLRP12, whereas the lung adenocarcinoma macrophage study suggests that elements of the C1qA/LILRB4/NF-κB circuit may be more immediately targetable than NLRP12 as a standalone molecule.

This downstream-circuit strategy has several advantages. First, many of the pathways linked to NLRP12 already have existing pharmacologic toolkits or are being actively explored therapeutically, including metabolic inhibitors, NF-κB-related modulators, myeloid-targeting strategies, and pathway-specific inhibitors of tumor signaling. Second, targeting the dominant output pathway may avoid some of the systemic consequences of directly altering an innate immune regulator with tissue-specific protective functions. Third, because NLRP12 may function through protein–protein interactions or scaffold-like mechanisms that are difficult to drug directly, focusing on tractable downstream effectors may offer a faster path to translation (93–95). In this sense, NLRP12 may initially be most useful not as a direct target, but as a biological compass that identifies which signaling or ecosystemal dependencies deserve therapeutic intervention.

### 9.3 NLRP12 and combination therapy opportunities

The context-dependent roles of NLRP12 also make it an attractive candidate for combination-based therapeutic reasoning (96). In macrophage-rich, immune-suppressed tumors, NLRP12-associated signaling may help explain why T-cell-directed immunotherapies alone are insufficient, suggesting that reprogramming the myeloid compartment could be necessary to unlock better antitumor responses. In metabolically adaptive tumors such as gastric cancer, NLRP12-driven glycolysis and lactylation may create vulnerabilities to metabolic interventions that could be paired with standard therapies or immunotherapy. Meanwhile, in settings where NLRP12 loss drives Wnt/β-catenin or JNK hyperactivation, patients might benefit more from pathway-matched combinations than from any attempt to target NLRP12 directly. These scenarios remain largely hypothesis-generating at present, but they provide a strong conceptual rationale for integrating NLRP12 into combination therapy design rather than considering it in isolation. An additional future avenue lies in cell-death-oriented strategies. Because HCK has been identified as a regulator of NLRP12-mediated PANoptosis, it is conceivable that NLRP12-linked death programs could someday be exploited to enhance tumor cell elimination or overcome resistance to apoptosis-dominant therapies. At present, however, this remains an early-stage concept. The available PANoptosis data establish mechanistic relevance for NLRP12 in integrated inflammatory cell death, but they do not yet define how to activate or modulate this pathway safely and effectively in cancer patients. Thus, PANoptosis-related applications should be framed as a forward-looking opportunity rather than a current therapeutic reality.

### 9.4 Current limitations and methodological challenges

Despite recent progress, several important limitations continue to restrict the interpretation of NLRP12 biology in cancer. First, lineage-specific mechanistic studies remain limited. Many available studies do not clearly distinguish whether NLRP12 acts in malignant epithelial cells, hepatocytes, macrophages, stromal cells, or other tumor-associated compartments. This limitation is especially important because tumor-cell-intrinsic NLRP12 may suppress oncogenic signaling, whereas myeloid-cell-associated NLRP12 may promote immunosuppressive remodeling. Second, bulk-tumor expression data are difficult to interpret. Increased or decreased NLRP12 expression in bulk RNA-seq datasets may reflect changes in tumor cells, immune-cell infiltration, stromal abundance, or inflammatory status rather than a true change in malignant-cell-intrinsic NLRP12 activity (97). Therefore, prognostic associations based on bulk expression should be interpreted cautiously and should not be assumed to indicate direct tumor-cell function. Third, single-cell and spatial validation remain insufficient. Although transcriptomic deconvolution studies suggest links between NLRP12 and immune infiltration, they cannot define the precise cellular source, spatial niche, or cell–cell interaction network involving NLRP12 (98, 99). Fourth, the cell-type-specific functions of NLRP12 remain incompletely understood. In particular, it is still unclear whether NLRP12 exerts distinct effects in tumor cells, tumor-associated macrophages, dendritic cells, fibroblasts, endothelial cells, or lymphocytes across different cancer types. Addressing these limitations will require lineage-specific perturbation models, single-cell RNA sequencing, spatial transcriptomics, multiplex immunostaining, organoid–immune co-culture systems, and prospective clinical cohorts with matched molecular and treatment-response data.

### 9.5 Key unresolved questions

Several major questions must be addressed before NLRP12 can be translated into clinically meaningful strategies. The first is cellular resolution: which cell type is functionally responsible for the NLRP12 signal in a given tumor? Bulk expression data are insufficient for this purpose, especially in tumors with abundant macrophage or stromal infiltration. The second is mechanistic prioritization: when NLRP12 is dysregulated, which downstream axis is actually driving the phenotype in that cancer context: NF-κB, Wnt/β-catenin, JNK, glycolysis, immune suppression, or PANoptosis-related signaling? The third is therapeutic directionality: should NLRP12 activity be preserved, inhibited, bypassed, or simply used for stratification? Without answering these questions, any attempt to generalize NLRP12-targeted therapy would be premature. A fourth unresolved issue is biomarker architecture. NLRP12 may ultimately prove more valuable as part of a composite framework than as a standalone marker. For example, its interpretation may require simultaneous assessment of macrophage abundance, glycolytic state, lactate-associated chromatin changes, Wnt/β-catenin activity, or JNK signaling depending on tumor type. In that case, the most useful clinical application of NLRP12 may be as one dimension within a layered ecosystem signature rather than as a single threshold-based assay. This possibility fits well with the broader view that NLRP12 functions as a context-sensitive organizer of tumor biology rather than a simple on/off regulator.

### 9.6 Experimental priorities for the next phase of the field

Future progress will depend on more precise and hypothesis-driven experimental strategies. First, single-cell RNA sequencing and spatial transcriptomics should be used to define the cellular source of NLRP12 expression and its spatial relationship with tumor cells, macrophages, T cells, stromal cells, hypoxic niches, and metabolically active regions within the tumor microenvironment. Second, lineage-specific knockout or knock-in models will be required to distinguish tumor-cell-intrinsic functions of NLRP12 from macrophage- or stromal-dependent effects. For example, epithelial/tumor-cell-specific and myeloid-cell-specific NLRP12 perturbation models could directly test whether NLRP12 suppresses oncogenic signaling in malignant cells while promoting immunosuppressive programs in tumor-associated macrophages (33, 64). Third, organoid-based co-culture systems incorporating macrophages, T cells, fibroblasts, or endothelial cells could be used to model tumor–immune reciprocity and evaluate how NLRP12 influences immune suppression, cytotoxic T-cell activity, and response to immunotherapy. Fourth, integrated phosphoproteomics, metabolomics, and epigenomic profiling should be applied to determine whether NLRP12 primarily regulates signaling pathways, glycolytic rewiring, lactate accumulation, histone lactylation, or PANoptosis-related stress responses in specific cancer contexts. Finally, prospective clinical cohorts with matched bulk transcriptomic, single-cell, spatial, and treatment-response data will be necessary to validate the prognostic and predictive value of NLRP12. These experimental strategies would allow future studies to move beyond correlative observations and directly test the context-dependent hypotheses proposed in this review.

Translationally, future studies should also move beyond retrospective association and test whether NLRP12 status can prospectively guide therapy selection or predict response. This will require well-annotated cohorts, standardized detection strategies, and ideally intervention-linked biomarker analyses. In parallel, the PANoptosis field may open new directions for exploring whether NLRP12-linked stress responses can be therapeutically engaged in cancer. Taken together, the next phase of NLRP12 research should aim not simply to catalog more associations, but to build a cell-resolved, pathway-aware, and therapeutically testable framework that explains when NLRP12 is protective, when it is harmful, and how that distinction can be exploited. The major unresolved questions, translational challenges, and priority directions for future NLRP12 research in cancer are outlined in **Table 3**. Preclinical safety studies should also evaluate whether NLRP12-targeted therapies perturb tumor-suppressive signaling in normal or premalignant tissues, particularly in organs where NLRP12 restrains inflammation-associated tumorigenesis.

**Table 3:** Outstanding questions, translational challenges, and future directions for NLRP12 research in cancer.

Unresolved issue	Why it matters	Current gap	Recommended strategy
What determines whether NLRP12 is tumor-suppressive or tumor-promoting?	Central to biological interpretation and therapeutic direction	Contradictory roles across CRC, HCC, GC, OV, glioma, and TAM-rich tumors	Perform lineage-resolved and tumor-type-specific mechanistic studies
Which cell type is the dominant source of NLRP12 in each tumor?	Tumor-cell versus macrophage-derived NLRP12 may imply opposite biology	Bulk RNA/IHC often cannot distinguish cellular origin	Use single-cell RNA-seq, spatial transcriptomics, multiplex IF/IHC
Which pathway is functionally dominant downstream of NLRP12?	Different tumors rely on different axes: NF-κB, Wnt/β-catenin, JNK, glycolysis, PANoptosis	Most studies focus on one pathway in one cancer type	Integrate phosphoproteomics, transcriptomics, metabolomics, and perturbation assays
Can NLRP12 predict treatment response?	Needed for clinical utility	Only preliminary evidence exists, especially in ovarian cancer	Validate in prospective cohorts and treatment-linked biomarker studies
Should NLRP12 itself be targeted, or should downstream circuits be targeted instead?	Direct targeting may be risky because NLRP12 can be protective in some tissues	No consensus on directionality of intervention	Prioritize pathway-centered targeting: e.g., β-catenin, JNK, HK2/glycolysis, LILRB4/NF-κB
How does NLRP12 intersect with tumor immunity?	May affect macrophage polarization and T-cell dysfunction	Causal links are strongest in LUAD TAMs but limited elsewhere	Develop co-culture, organoid–immune, and myeloid-specific knockout models
How broad is the metabolic role of NLRP12?	Could expand NLRP12 from immune regulator to metabolic adaptor	Strong evidence mainly exists in gastric cancer	Test NLRP12 effects on glycolysis, lactate flux, mitochondrial stress, and chromatin remodeling in other tumors
Is NLRP12-linked PANoptosis therapeutically exploitable in cancer?	Could open new cell-death-based strategies	Current PANoptosis evidence is largely non-cancer-specific	Assess tumor-cell and immune-cell PANoptotic responses in therapy models
How should NLRP12 be incorporated clinically?	Practical translation requires assay standardization	No standardized cutoff, platform, or workflow	Build tumor-specific composite panels including NLRP12 plus pathway/immune markers

## 10 Conclusions

NLRP12 should no longer be viewed solely as an inflammasome-associated anti-inflammatory molecule. Current evidence indicates that its role in cancer varies according to tumor lineage, cellular compartment, dominant signaling pathway, and microenvironmental state. In colorectal cancer, hepatocellular carcinoma, and triple-negative breast cancer, NLRP12 mainly acts as a tumor-suppressive checkpoint by restraining noncanonical NF-κB, Wnt/β-catenin, JNK, or canonical NF-κB signaling, thereby limiting inflammation-driven tumorigenesis, proliferation, and invasion. In contrast, in gastric cancer, ovarian cancer, glioma, and macrophage-rich tumor microenvironments, NLRP12 may contribute to tumor progression through glycolytic reprogramming, lactate-associated epigenetic remodeling, aggressive phenotypes, or myeloid-mediated immune suppression. Thus, the dual role of NLRP12 is best explained by its cellular source and pathway context rather than by a fixed tumor-suppressive or oncogenic identity. This context-specific interpretation has important clinical implications. NLRP12 may serve as a useful biomarker or therapeutic reference point only when its tumor type, cellular origin, and dominant downstream circuit are clearly defined. Future studies using single-cell, spatial, genetic, metabolic, and prospective clinical approaches will be needed to determine when NLRP12 should be restored, inhibited, or used for patient stratification. Such work may help transform NLRP12 from an underappreciated innate immune regulator into a more precise tool for understanding tumor biology and guiding cancer therapy.

## References

[1] Fonseca G Farkas J Dora E von Haehling S Lainscak M . Cancer cachexia and related metabolic dysfunction. Int J Mol Sci. (2020) 21. doi: 10.3390/ijms21072321 32230855 PMC7177950

[2] Iaia N Noviello C Muscaritoli M Costelli P . Inflammation in cancer cachexia: still the central tenet or just another player? Am J Physiol Cell Physiol. (2025) 328:C1837–c1852. doi: 10.1152/ajpcell.00808.2024 40250836

[3] Tsoli M Robertson G . Cancer cachexia: Malignant inflammation, tumorkines, and metabolic mayhem. Trends Endocrinol Metab. (2013) 24:174–83. doi: 10.1016/j.tem.2012.10.006 23201432

[4] Ge T Gu X Jia R Ge S Chai P Zhuang A . Crosstalk between metabolic reprogramming and epigenetics in cancer: updates on mechanisms and therapeutic opportunities. Cancer Commun (Lond). (2022) 42:1049–82. doi: 10.1002/cac2.12374 36266736 PMC9648395

[5] Kroemer G Pouyssegur J . Tumor cell metabolism: cancer's Achilles' heel. Cancer Cell. (2008) 13:472–82. doi: 10.1016/j.ccr.2008.05.005 18538731

[6] Sundaram B Tweedell RE Prasanth Kumar S Kanneganti TD . The NLR family of innate immune and cell death sensors. Immunity. (2024) 57:674–99. doi: 10.1016/j.immuni.2024.03.012 38599165 PMC11112261

[7] Sharma N Jha S . NLR-regulated pathways in cancer: opportunities and obstacles for therapeutic interventions. Cell Mol Life Sci CMLS. (2016) 73:1741–64. doi: 10.1007/s00018-015-2123-8 26708292 PMC11108278

[8] Wilmanski JM Petnicki-Ocwieja T Kobayashi KS . NLR proteins: integral members of innate immunity and mediators of inflammatory diseases. J Leukocyte Biol. (2008) 83:13–30. doi: 10.1189/jlb.0607402 17875812 PMC3256237

[9] Huang L Tao Y Wu X Wu J Shen M Zheng Z . The role of NLRP12 in inflammatory diseases. Eur J Pharmacol. (2023) 956:175995. doi: 10.1016/j.ejphar.2023.175995 37572944

[10] Zaki MH Vogel P Malireddi RK Body-Malapel M Anand PK Bertin J . The NOD-like receptor NLRP12 attenuates colon inflammation and tumorigenesis. Cancer Cell. (2011) 20:649–60. doi: 10.1016/j.ccr.2011.10.022 22094258 PMC3761879

[11] Zhou L Wang Z Huang Y Zhang X Jiang H Guo Z . NLRP12 decreases TRIM25-mediated HK2 degradation to promote glycolysis and H3K18la in gastric cancer. Cell Death Dis. (2025) 16:615. doi: 10.1038/s41419-025-07923-3 40796546 PMC12343871

[12] Sharma N Saxena S Agrawal I Singh S Srinivasan V Arvind S . Differential expression profile of NLRs and AIM2 in glioma and implications for NLRP12 in glioblastoma. Sci Rep. (2019) 9:8480. doi: 10.1038/s41598-019-44854-4 31186453 PMC6559951

[13] Nadendla EK Alluri P Sundaram B Kumar SP Chadchan SB Sarkar R . HCK regulates NLRP12-mediated PANoptosis. PNAS. (2025) 122:e2422079122. doi: 10.1073/pnas.2422079122 40408404 PMC12130821

[14] Pinheiro AS Eibl C Ekman-Vural Z Schwarzenbacher R Peti W . The NLRP12 pyrin domain: structure, dynamics, and functional insights. J Mol Biol. (2011) 413:790–803. doi: 10.1016/j.jmb.2011.09.024 21978668 PMC3202057

[15] Jin T Huang M Jiang J Smith P Xiao TS . Crystal structure of human NLRP12 PYD domain and implication in homotypic interaction. PloS One. (2018) 13:e0190547. doi: 10.1371/journal.pone.0190547 29293680 PMC5749810

[16] Morrison HA Trusiano B Rowe AJ Allen IC . Negative regulatory NLRs mitigate inflammation via NF-κB pathway signaling in inflammatory bowel disease. BioMed J. (2023) 46:100616. doi: 10.1016/j.bj.2023.100616 37321320 PMC10494316

[17] Thaiss CA Elinav E . NF-κB regulation by NLRs: T cells join the club. Immunity. (2015) 42:595–7. doi: 10.1016/j.immuni.2015.03.010 25902475

[18] Tengesdal IW Dinarello CA Marchetti C . NLRP3 and cancer: pathogenesis and therapeutic opportunities. Pharmacol Ther. (2023) 251:108545. doi: 10.2139/ssrn.4501298 37866732 PMC10710902

[19] Sharma BR Kanneganti TD . NLRP3 inflammasome in cancer and metabolic diseases. Nat Immunol. (2021) 22:550–9. doi: 10.1038/s41590-021-00886-5 33707781 PMC8132572

[20] Khan A Alzahrani AR Jawaid T Al-Bazi MM Banjabi AA Hai A . SLC7A11 and NLRP1 in non-small cell lung cancer. Clin Chim Acta. (2026) 579:120684. doi: 10.1016/j.cca.2025.120684 41192535

[21] Yan J Xu W Lenahan C Huang L Wen J Li G . CCR5 activation promotes NLRP1-dependent neuronal pyroptosis via CCR5/PKA/CREB pathway after intracerebral hemorrhage. Stroke. (2021) 52:4021–32. doi: 10.1161/strokeaha.120.033285 34719258 PMC8607924

[22] Navuluri N Yata VK Dachani SR Rachakonda K Kolliputi N . Heme unleashed: NLRP12 orchestrates PANoptosis in a symphony of cell fate. Cell Biochem Biophys. (2025) 83:2727–33. doi: 10.1007/s12013-025-01712-7 40055236

[23] Sharma BR Karki R Rajesh Y Kanneganti TD . Immune regulator IRF1 contributes to ZBP1-, AIM2-, RIPK1-, and NLRP12-PANoptosome activation and inflammatory cell death (PANoptosis). J Biol Chem. (2023) 299:105141. doi: 10.1016/j.jbc.2023.105141 37557956 PMC10494469

[24] Udden SN Kwak YT Godfrey V Khan MAW Khan S Loof N . NLRP12 suppresses hepatocellular carcinoma via downregulation of cJun N-terminal kinase activation in the hepatocyte. Elife. (2019) 8. doi: 10.7554/elife.40396 30990169 PMC6483596

[25] Khan S Zaki H . Crosstalk between NLRP12 and JNK during hepatocellular carcinoma. Int J Mol Sci. (2020) 21. doi: 10.3390/ijms21020496 31941025 PMC7013925

[26] Pandeya A Kanneganti TD . Therapeutic potential of PANoptosis: innate sensors, inflammasomes, and RIPKs in PANoptosomes. Trends Mol Med. (2024) 30:74–88. doi: 10.1016/j.molmed.2023.10.001 37977994 PMC10842719

[27] Pu X Wu Y Peng C Sun X Zuo H Yuan X . PANoptosis in cancer: molecular mechanisms and therapeutic potential. Cancer Gene Ther. (2025) 32:1042–53. doi: 10.1038/s41417-025-00940-6 40841819

[28] Sun X Yang Y Meng X Li J Liu X Liu H . PANoptosis: mechanisms, biology, and role in disease. Immunol Rev. (2024) 321:246–62. doi: 10.1111/imr.13279 37823450

[29] Wang WQ Zhou Z Ge FX Tayir M Hao MY Wu DD . Role of PANoptosis in cancer: molecular mechanisms and therapeutic opportunities. Apoptosis. (2025) 30:2722–44. doi: 10.1007/s10495-025-02173-2 41006688

[30] Allen IC Wilson JE Schneider M Lich JD Roberts RA Arthur JC . NLRP12 suppresses colon inflammation and tumorigenesis through the negative regulation of noncanonical NF-κB signaling. Immunity. (2012) 36:742–54. doi: 10.1016/j.immuni.2012.03.012 22503542 PMC3658309

[31] Pudla M Onsoi P Utaisincharoen P . NLRP12 attenuates tumor necrosis factor-α production in Burkholderia pseudomallei-infected RAW264.7 macrophages. Asian Pac J Allergy Immunol. (2025) 43:727–31. doi: 10.12932/AP-210422-1373 36278779

[32] Pudla M Srisaowakarn C Utaisincharoen P . NLRP12 negatively modulates inducible nitric oxide synthase (iNOS) expression and tumor necrosis factor-α production in Porphyromonas gingivalis LPS-treated mouse macrophage cell line (RAW264.7). Inflammation Res. (2019) 68:841–4. doi: 10.1007/s00011-019-01267-3 31292668

[33] Rajkhowa S Jha S . The role of NLRP3 and NLRP12 inflammasomes in glioblastoma. Genes Immun. (2024) 25:541–51. doi: 10.1038/s41435-024-00309-z 39604503

[34] Kuang W Gu Q Zhou Y Xiao X He D Deng Q . Inhibited expression of NLRP12 promotes the development of triple-negative breast cancer by activating the NF-κB pathway. Cell Biochem Biophys. (2023) 81:727–35. doi: 10.1007/s12013-023-01166-9 37658975 PMC10611651

[35] Asati V Mahapatra DK Bharti SK . PI3K/Akt/mTOR and Ras/Raf/MEK/ERK signaling pathways inhibitors as anticancer agents: structural and pharmacological perspectives. Eur J Med Chem. (2016) 109:314–41. doi: 10.1016/j.ejmech.2016.01.012 26807863

[36] Cao Z Liao Q Su M Huang K Jin J Cao D . AKT and ERK dual inhibitors: the way forward? Cancer Lett. (2019) 459:30–40. doi: 10.1016/j.canlet.2019.05.025 31128213

[37] Zhang Z Richmond A Yan C . Immunomodulatory properties of PI3K/AKT/mTOR and MAPK/MEK/ERK inhibition augment response to immune checkpoint blockade in melanoma and triple-negative breast cancer. Int J Mol Sci. (2022) 23. doi: 10.3390/ijms23137353 35806358 PMC9266842

[38] Khan S Kwak YT Peng L Hu S Cantarel BL Lewis CM . NLRP12 downregulates the Wnt/β-catenin pathway via interaction with STK38 to suppress colorectal cancer. J Clin Invest. (2023) 133. doi: 10.1172/jci166295 37581937 PMC10541192

[39] Cao X Li L Hu J Zhu S Song S Kong S . Neohesperidin protects against colitis-associated colorectal cancer in mice via suppression of the NF-κB/p65 and MAPK pathways. J Nutr Biochem. (2025) 136:109804. doi: 10.1016/j.jnutbio.2024.109804 39547268

[40] Korbecki J Simińska D Gąssowska-Dobrowolska M Listos J Gutowska I Chlubek D . Chronic and cycling hypoxia: drivers of cancer chronic inflammation through HIF-1 and NF-κB activation: a review of the molecular mechanisms. Int J Mol Sci. (2021) 22. doi: 10.3390/ijms221910701 34639040 PMC8509318

[41] Liu X Li Y Yuan C Zhao Y Zhou L Yan Y . Sophocarpine suppresses MAPK-mediated inflammation by restoring gut microbiota in colorectal cancer. Phytomedicine. (2025) 143:156833. doi: 10.1016/j.phymed.2025.156833 40393246

[42] Zou F Mao R Yang L Lin S Lei K Zheng Y . Targeted deletion of miR-139-5p activates MAPK, NF-κB and STAT3 signaling and promotes intestinal inflammation and colorectal cancer. FEBS J. (2016) 283:1438–52. doi: 10.1111/febs.13678 26859226

[43] Zununi Vahed S Barzegari A Rahbar Saadat Y Goreyshi A Omidi Y . Leuconostoc mesenteroides-derived anticancer pharmaceuticals hinder inflammation and cell survival in colon cancer cells by modulating NF-κB/AKT/PTEN/MAPK pathways. BioMed Pharmacother. (2017) 94:1094–100. doi: 10.1016/j.biopha.2017.08.033 28821160

[44] Arrè V Negro R Giannelli G . The role of inflammasomes in hepatocellular carcinoma: mechanisms and therapeutic insights. Ann Hepatol. (2025) 30:101772. doi: 10.1016/j.aohep.2024.101772 39701280

[45] Ali M Bamezai RNK Singh RP . Invasive breast cancer: miR-24-2 targets genes associated with survival and sensitizes MDA-MB-231 cells to berberine. Omics. (2023) 27:409–20. doi: 10.1089/omi.2023.0092 37669117

[46] Chauhan G Pathak DP Ali F Dubey P Khasimbi S . In-vitro evaluation of isatin derivatives as potent anti-breast cancer agents against MCF-7, MDA MB 231, MDA-MB 435 and MDA-MB 468 breast cancers cell lines: a review. Anti-Cancer Agents Med Chem. (2022) 22:1883–96. doi: 10.2174/1871520621666210903130152 34477529

[47] Lyu ZY Zhang XY Chen H Jiang PR Chen JY . Effects of SmacN7 inducing apoptosis of breast cancer cell MDA-MB-157 and its mechanism. Zhongguo Ying Yong Sheng Li Xue Za Zhi. (2020) 36:642–7. doi: 10.12047/j.cjap.5948.2020.134 33719274

[48] Wang W Chang S He X Zhou X Shang P Chen Y . Sulforaphane inhibits the migration and invasion of BPDE-induced lung adenocarcinoma cells by regulating NLRP12. Toxicol Appl Pharmacol. (2024) 485:116916. doi: 10.1016/j.taap.2024.116916 38537874

[49] Sebio A Kahn M Lenz HJ . The potential of targeting Wnt/β-catenin in colon cancer. Expert Opin Ther Targets. (2014) 18:611–5. doi: 10.1517/14728222.2014.906580 24702624

[50] Tewari D Bawari S Sharma S DeLiberto LK Bishayee A . Targeting the crosstalk between canonical Wnt/β-catenin and inflammatory signaling cascades: a novel strategy for cancer prevention and therapy. Pharmacol Ther. (2021) 227:107876. doi: 10.1016/j.pharmthera.2021.107876 33930452

[51] Vallée A Lecarpentier Y Vallée JN . Targeting the canonical WNT/β-catenin pathway in cancer treatment using non-steroidal anti-inflammatory drugs. Cells. (2019) 8. doi: 10.3390/cells8070726 PMC6679009 31311204

[52] Zhao Q Bi Y Zhong J Ren Z Liu Y Jia J . Pristimerin suppresses colorectal cancer through inhibiting inflammatory responses and Wnt/β-catenin signaling. Toxicol Appl Pharmacol. (2020) 386:114813. doi: 10.1016/j.taap.2019.114813 31715269

[53] Chiang C Yap BK . TRIM25, TRIM28 and TRIM59 and their protein partners in cancer signaling crosstalk: Potential novel therapeutic targets for cancer. Curr Issues Mol Biol. (2024) 46:10745–61. doi: 10.3390/cimb46100638 39451518 PMC11506413

[54] Dang T You Y Wei L Li Q Sun H Sun M . ICAT drives lactylation of tumor-associated macrophages via the c-Myc-ENO1 axis to promote cervical cancer progression. Free Radic Biol Med. (2025) 241:316–29. doi: 10.1016/j.freeradbiomed.2025.09.031 40976412

[55] Zhang C Zhou L Zhang M Du Y Li C Ren H . H3K18 lactylation potentiates immune escape of non-small cell lung cancer. Cancer Res. (2024) 84:3589–601. doi: 10.1158/0008-5472.can-23-3513 39137401

[56] Ma R Tang Z Wang J . NLRP12 is a prognostic biomarker and correlated with immune infiltrates in epithelial ovarian cancer. J Gene Med. (2024) 26:e3585. doi: 10.1002/jgm.3585 37926491

[57] Cheng YW Chen YY Lin CJ Chen YT Lieu AS Tsai HP . High expression of NLRP12 predicts poor prognosis in patients with intracranial glioma. J Chin Med Assoc. (2023) 86:88–97. doi: 10.1097/jcma.0000000000000830 36599143 PMC12755504

[58] Cemeli T Guasch-Vallés M Nàger M Felip I Cambray S Santacana M . Cytoplasmic cyclin D1 regulates glioblastoma dissemination. J Pathol. (2019) 248:501–13. doi: 10.1002/path.5277 30957234

[59] Miletic H Niclou SP Johansson M Bjerkvig R . Anti-VEGF therapies for Malignant glioma: treatment effects and escape mechanisms. Expert Opin Ther Targets. (2009) 13:455–68. doi: 10.1517/14728220902806444 19335067

[60] Seyedmirzaei H Shobeiri P Turgut M Hanaei S Rezaei N . VEGF levels in patients with glioma: a systematic review and meta-analysis. Rev Neurosci. (2021) 32:191–202. doi: 10.1515/revneuro-2020-0062 33125340

[61] Noh MG Oh SJ Ahn EJ Kim YJ Jung TY Jung S . Prognostic significance of E-cadherin and N-cadherin expression in gliomas. BMC Cancer. (2017) 17:583. doi: 10.1186/s12885-017-3591-z 28851312 PMC5575836

[62] El Hassouni B Granchi C Vallés-Martí A Supadmanaba IGP Bononi G Tuccinardi T . The dichotomous role of the glycolytic metabolism pathway in cancer metastasis: Interplay with the complex tumor microenvironment and novel therapeutic strategies. Semin Cancer Biol. (2020) 60:238–48. doi: 10.1016/j.semcancer.2019.08.025 31445217

[63] Wang D Guan H . Glycolytic reprogramming in macrophage polarization: New horizons in the treatment of tumor diseases. Cell Signalling. (2025) 134:111940. doi: 10.1016/j.cellsig.2025.111940 40553967

[64] Yin J Song Y Fu Y Wang J Zhang Z Ruan S . NLRP12/C1qA positive feedback in tumor-associated macrophages regulates immunosuppression through LILRB4/NF-κB pathway in lung adenocarcinoma. Cancer Immunol Immunother CII. (2024) 74:16. doi: 10.1007/s00262-024-03880-6 39527158 PMC11554950

[65] Li Y Shen Z Chai Z Zhan Y Zhang Y Liu Z . Targeting MS4A4A on tumour-associated macrophages restores CD8+ T-cell-mediated antitumour immunity. Gut. (2023) 72:2307–20. doi: 10.1136/gutjnl-2022-329147 37507218 PMC10715532

[66] Rodriguez-Garcia A Lynn RC Poussin M Eiva MA Shaw LC O'Connor RS . CAR-T cell-mediated depletion of immunosuppressive tumor-associated macrophages promotes endogenous antitumor immunity and augments adoptive immunotherapy. Nat Commun. (2021) 12:877. doi: 10.1038/s41467-021-20893-2 33563975 PMC7873057

[67] Ramirez CFA Akkari L . Myeloid cell path to Malignancy: Insights into liver cancer. Trends Cancer. (2025) 11:591–610. doi: 10.1016/j.trecan.2025.02.006 40140328

[68] Sica A Massarotti M . Myeloid suppressor cells in cancer and autoimmunity. J Autoimmun. (2017) 85:117–25. doi: 10.1016/j.jaut.2017.07.010 28728794

[69] Sivagnanalingam U Beatty PL Finn OJ . Myeloid derived suppressor cells in cancer, premalignancy and inflammation: A roadmap to cancer immunoprevention. Mol Carcinog. (2020) 59:852–61. doi: 10.1002/mc.23206 32333615

[70] Szebeni GJ Vizler C Nagy LI Kitajka K Puskas LG . Pro-tumoral inflammatory myeloid cells as emerging therapeutic targets. Int J Mol Sci. (2016) 17. doi: 10.3390/ijms17111958 27886105 PMC5133952

[71] Zeng W Liu H Mao Y Jiang S Yi H Zhang Z . Myeloid-derived suppressor cells: Key immunosuppressive regulators and therapeutic targets in colorectal cancer (Review). Int J Oncol. (2024) 65. doi: 10.3892/ijo.2024.5673 39054950 PMC11299769

[72] Kumar S Tailor D Dheeraj A Li W Stefan K Lee JM . Uncovering therapeutic targets for macrophage-mediated T cell suppression and PD-L1 therapy sensitization. Cell Rep Med. (2024) 5:101698. doi: 10.1016/j.xcrm.2024.101698 39181134 PMC11524979

[73] Loeuillard E Yang J Buckarma E Wang J Liu Y Conboy C . Targeting tumor-associated macrophages and granulocytic myeloid-derived suppressor cells augments PD-1 blockade in cholangiocarcinoma. J Clin Invest. (2020) 130:5380–96. doi: 10.1172/jci137110 32663198 PMC7524481

[74] Antonangeli F Natalini A Garassino MC Sica A Santoni A Di Rosa F . Regulation of PD-L1 expression by NF-κB in cancer. Front Immunol. (2020) 11:584626. doi: 10.3389/fimmu.2020.584626 33324403 PMC7724774

[75] de la Calle-Fabregat C Calafell-Segura J Gardet M Dunsmore G Mulder K Ciudad L . NF-κB and TET2 promote macrophage reprogramming in hypoxia that overrides the immunosuppressive effects of the tumor microenvironment. Sci Adv. (2024) 10:eadq5226. doi: 10.1126/sciadv.adq5226 39292770 PMC11409945

[76] Gu M Zhou X Sohn JH Zhu L Jie Z Yang JY . NF-κB-inducing kinase maintains T cell metabolic fitness in antitumor immunity. Nat Immunol. (2021) 22:193–204. doi: 10.1038/s41590-020-00829-6 33398181 PMC7855506

[77] Ye Z Lich JD Moore CB Duncan JA Williams KL Ting JP . ATP binding by monarch-1/NLRP12 is critical for its inhibitory function. Mol Cell Biol. (2008) 28:1841–50. doi: 10.1128/mcb.01468-07 18160710 PMC2258772

[78] Liang P Li Z Chen Z Chen Z Jin T He F . Metabolic reprogramming of glycolysis, lipids, and amino acids in tumors: Impact on CD8+ T cell function and targeted therapeutic strategies. FASEB J. (2025) 39:e70520. doi: 10.1096/fj.202403019r 40249661

[79] Bandopadhyay S Kamal IM Padmanaban E Ghosh DD Chakrabarti S Roy SS . Oncogene-mediated nuclear accumulation of lactate promotes epigenetic alterations to induce cancer cell proliferation. J Cell Biochem. (2023) 124:495–519. doi: 10.1002/jcb.30381 36999756

[80] Pucino V Cucchi D Mauro C . Lactate transporters as therapeutic targets in cancer and inflammatory diseases. Expert Opin Ther Targets. (2018) 22:735–43. doi: 10.1080/14728222.2018.1511706 30106309

[81] San-Millán I Brooks GA . Reexamining cancer metabolism: Lactate production for carcinogenesis could be the purpose and explanation of the Warburg effect. Carcinogenesis. (2017) 38:119–33. doi: 10.1093/carcin/bgw127 27993896 PMC5862360

[82] Gatenby RA Gillies RJ . Why do cancers have high aerobic glycolysis? Nat Rev Cancer. (2004) 4:891–9. doi: 10.1038/nrc1478 15516961

[83] Wu Z Wu J Zhao Q Fu S Jin J . Emerging roles of aerobic glycolysis in breast cancer. Clin Transl Oncol. (2020) 22:631–46. doi: 10.1007/s12094-019-02187-8 31359335

[84] Xie Y Wang M Xia M Guo Y Zu X Zhong J . Ubiquitination regulation of aerobic glycolysis in cancer. Life Sci. (2022) 292:120322. doi: 10.1016/j.lfs.2022.120322 35031261

[85] Karan D Tawfik O Dubey S . Expression analysis of inflammasome sensors and implication of NLRP12 inflammasome in prostate cancer. Sci Rep. (2017) 7:4378. doi: 10.1038/s41598-017-04286-4 28663562 PMC5491527

[86] Veeranki S . Role of inflammasomes and their regulators in prostate cancer initiation, progression and metastasis. Cell Mol Biol Lett. (2013) 18:355–67. doi: 10.2478/s11658-013-0095-y 23793845 PMC6275599

[87] Zhao M Guo J Gao QH Wang H Wang F Wang ZR . Relationship between pyroptosis-mediated inflammation and the pathogenesis of prostate disease. Front Med (Lausanne). (2023) 10:1084129. doi: 10.3389/fmed.2023.1084129 36744134 PMC9892550

[88] Wu H Liu B Chen Z Li G Zhang Z . MSC-induced lncRNA HCP5 drove fatty acid oxidation through miR-3619-5p/AMPK/PGC1α/CEBPB axis to promote stemness and chemo-resistance of gastric cancer. Cell Death Dis. (2020) 11:233. doi: 10.1038/s41419-020-2426-z 32300102 PMC7162922

[89] Gao L Shay C Teng Y . Cell death shapes cancer immunity: Spotlighting PANoptosis. J Exp Clin Cancer Res. (2024) 43:168. doi: 10.1186/s13046-024-03089-6 38877579 PMC11179218

[90] Lin JF Wang TT Huang RZ Tan YT Chen DL Ju HQ . PANoptosis in cancer: Bridging molecular mechanisms to therapeutic innovations. Cell Mol Immunol. (2025) 22:996–1011. doi: 10.1038/s41423-025-01329-z 40721869 PMC12398562

[91] Wang S Li Z Hou J Li X Ni Q Wang T . Integrating PANoptosis insights to enhance breast cancer prognosis and therapeutic decision-making. Front Immunol. (2024) 15:1359204. doi: 10.3389/fimmu.2024.1359204 38504988 PMC10948567

[92] Xie Z Yang T Zhou C Xue Z Wang J Lu F . Integrative bioinformatics analysis and experimental study of NLRP12 reveal its prognostic value and potential functions in ovarian cancer. Mol Carcinog. (2025) 64:383–98. doi: 10.1002/mc.23854 39601513

[93] Kim M Park J Bouhaddou M Kim K Rojc A Modak M . A protein interaction landscape of breast cancer. Science. (2021) 374:eabf3066. doi: 10.1126/science.abf3066 34591612 PMC9040556

[94] White AW Westwell AD Brahemi G . Protein-protein interactions as targets for small-molecule therapeutics in cancer. Expert Rev Mol Med. (2008) 10:e8. doi: 10.1017/s1462399408000641 18353193

[95] Yoon TY Lee HW . Shedding light on complexity of protein-protein interactions in cancer. Curr Opin Chem Biol. (2019) 53:75–81. doi: 10.1016/j.cbpa.2019.07.001 31479832

[96] Jéru I Hentgen V Normand S Duquesnoy P Cochet E Delwail A . Role of interleukin-1β in NLRP12-associated autoinflammatory disorders and resistance to anti-interleukin-1 therapy. Arthritis Rheum. (2011) 63:2142–8. doi: 10.1002/art.30378 21480187

[97] Ren X Kang B Zhang Z . Understanding tumor ecosystems by single-cell sequencing: Promises and limitations. Genome Biol. (2018) 19:211. doi: 10.1186/s13059-018-1593-z 30509292 PMC6276232

[98] Tirosh I . Pitfalls in analysis and interpretation of single-cell RNA-seq data in cancer. Neuro-oncol Adv. (2026) 8:i57–60. doi: 10.1093/noajnl/vdaf047 41769412 PMC12946769

[99] Dai Y Guo S Pan Y Castignani C Montierth MD Van Loo P . A guide to transcriptomic deconvolution in cancer. Nat Rev Cancer. (2026) 26:84–103. doi: 10.1038/s41568-025-00886-9 41331516 PMC7618897

